# Revisiting our primate roots in infants grooming

**DOI:** 10.1038/s41598-026-39909-2

**Published:** 2026-02-13

**Authors:** Chunmiao Mai, Guillaume Lio, Maude Beaudoin-Gobert, Chen Qu, Liuba Papeo, Michel Desmurget, Jean-Réné Duhamel, Irene Cristofori, Angela Sirigu

**Affiliations:** 1 Institute of Neuroscience la Timone UMR7289, Aix-Marseille University, Marseille, France; 2 Claude Bernard University, Villeurbanne, 69100 France; 3 iMIND Center of Excellence for Autism, Vinatier Hospital, Bron, France; 4 Department of Psychology, South China Normal University, Guangzhou, 510631 China; 5 Present Address: School of Educational Science, Lingnan Normal University, Zhanjiang, China

**Keywords:** Grooming, Non-human primate, Neuro-development, Language, Cognitive neuroscience, Social behaviour

## Abstract

Grooming is a common behavior across many animal species, including non-human primates (NHP), and serves a variety of functions, such as cleaning hair, alleviating stress, and enhancing social bonds. This study aims to explore whether grooming-like behaviors are present in humans during early developmental stages, before the emergence of gestural and language communication. Through the observations of 67 preverbal infants, we identified frequent manual behaviors, including grasping, holding, and behaviors resembling grooming, particularly directed toward caregivers’ hairy skin. These behaviors were analyzed and validated by twelve independent primatologists, who confirmed that behavioral sequences and their kinematics closely resembled grooming behaviors seen in NHPs, while also distinguishing them from other types of manual actions such as holding or grasping. Longitudinal analyses demonstrated a significant reduction in infants’ grooming behaviors beginning at 8 months, with these behaviors no longer observed by 15 months, a developmental shift that coincided with the emergence of more sophisticated gestural communication. Interestingly, grooming frequency, but not other actions, peaked during a specific time window, between 2pm and 4pm, which corresponds with a well-documented circadian dip in beta-endorphins, a neurochemical associated with stress regulation and social bonding. This alignment points toward a potential physiological underpinning for the timing of these behaviors. These findings suggest that infant grooming behavior represents a vestigial motor pattern, likely reflecting conserved evolutionary mechanisms shared with non-human primates. This behavior may represent a primitive form of early social interaction, highlighting the role of ancient motor programs in shaping prelinguistic communication.

**Supplementary Information:**

The online version contains supplementary material available at 10.1038/s41598-026-39909-2.

## Introduction

Grooming is a characteristic behavior in non-human primates (NHPs) which involves a range of coordinated hand and body movements. While the index finger and thumb are used to remove debris or ectoparasites from hair, NHP also employ sweeping motions of the hand, gentle parting of the hair with both hands, and close visual inspection of the skin. These actions are often accompanied by subtle body shifts to maintain balance and social contact^1^. Monkeys and great apes groom each other daily, a widespread behavior emerging with the development of precision grip^2–5^. This behavior serves multiple purposes: it helps maintain hygiene and plays a crucial role in managing stress and anxiety^6–9^. When grooming occurs between individuals, it also helps establish social hierarchies and strengthens social bonds, which can influence access to resources such as food, mating opportunities, and social support^10–13^.

Grooming behaviors are distinctive both behaviorally but also physiologically, activating C-afferent tactile receptors and stimulating endorphin release, thereby enhancing social bonding and reward^14,15^. In humans, while various interactive behaviors involving touch, such as washing hair, giving massages, or cleaning wounds, can fulfill similar social functions^16–18^they lack the specific motor action patterns seen in NHP grooming. Consequently, despite the prevalence of grooming in monkey and great ape social groups, evolution does not appear to have preserved or reinforced this particular behavioral trait in humans.

Evolutionary psychology theories, most notably advanced by Dunbar^1^, suggest that the decline of grooming as a primary social bonding mechanism in humans stems from both anatomical and cognitive developments. Biologically, the evolution of relatively hairless, smoother skin reduced the necessity of grooming for hygiene, as humans became less prone to parasite and debris accumulation compared with other primates. Socio-cognitively, the emergence of language is thought to have replaced grooming by offering a more flexible and efficient means of sustaining social cohesion. While grooming in nonhuman primates’ functions as a dyadic, *tactile* form of communication, limited by physical proximity and small group size, language enables the simultaneous exchange of information with multiple individuals, thereby supporting social bonding on a much larger scale. This shift is considered central to the expansion of human social networks and the evolution of complex, cooperative social structures^1,8,16^. On a phylogenetic timescale, then, the decline of grooming in humans appears to coincide with the development of symbolic gestures and language. Yet, it may be argued that grooming motor programs remain hardwired in the human brain and can surface during early neurodevelopment. Indeed, research on sensorimotor cortex stimulation during infant surgeries has revealed innate motor synergies, for example, coordinated hand-to-mouth movements accompanied by anticipatory mouth opening, suggesting the preservation of ancestral action patterns with ethological value¹^[,7^. This raises a provocative question: could the ontogeny of an infant reveal both the emergence and decline of grooming behavior?

In view of the above considerations, we propose, two contrasting hypotheses, regarding the emergence of social grooming in human infants. The *extinction hypothesis* argues that grooming behavior has been lost from the innate human motor and social repertoire through evolutionary change because it does not receive consistent environmental support or feedback that would typically strengthen, regulate, or stabilize it over time. As a result, grooming is not expected to appear at any point in human development, including infancy. According to this view, grooming no longer serves as a functional or observable behavior in early human social interaction. In contrast, the *developmental reactivation hypothesis* suggests that although grooming may not be consistently expressed throughout development, the underlying neural pathways involved in early grooming are preserved and may lie dormant. These pathways can be reactivated during a specific window in infancy, particularly before the emergence of symbolic gestural or verbal communication. During this period, infants typically engage in manipulative behaviors such as reaching, pinching, or holding. The hypothesis proposes that grooming behaviors may transiently re-emerge during this stage, reflecting a brief but meaningful reactivation of ancestral or early developmental systems for social touch and bonding.

To explore these hypotheses, we recorded infants’ behavior in both a controlled laboratory setting and through remote, naturalistic video observations in their homes. This dual approach enabled a structured investigation while establishing the preservation of spontaneous grooming behavior in a familiar environment. As a first step, we developed an ethogram to generate a comprehensive list of infant behaviors observed during the testing sessions. To specifically characterize the distinctive motor features of grooming behavior, our primary analyses focused on a subset of manual behaviors selected for the purposes of this study.

## Results

**Fig. 1:**
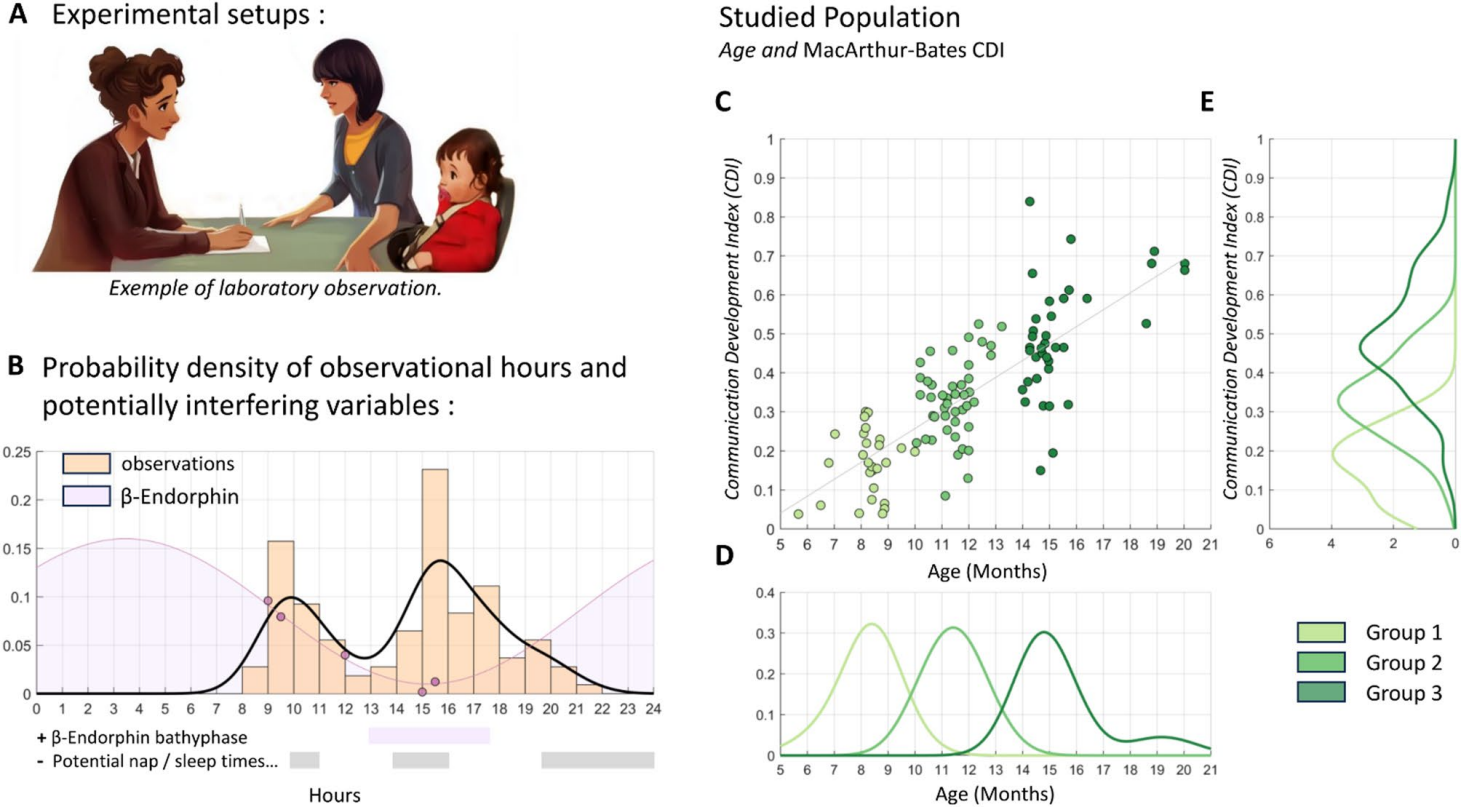
Test Setting and Population Characteristics. (**A**) The experimental setup in the laboratory. A similar spatial arrangement was designed during the video call condition. We recorded the entire session for offline coding analysis. In total, we collected 48 observations (*N* = 41 infants) in the lab and 60 observations (*N* = 26 infants) at the parents’ homes; (**B**) Distribution of observation hours for both laboratory and video call conditions. ^22,23,6,24^ (**C)** Infants’ neurodevelopmental scores, on MacArthur–Bates Communicative Development Inventories (CDI) plotted against infants’ age. ; (**D**) Due to recruitment factors, such as testing periods and intervals between exams, the age distribution is not continuous uniform, but can be best explained by three Gaussian distributions. Analyzing the data within these three age categories therefore provides a simpler and more appropriate approach. The distributions show smoothed theoretical estimates for each group, calculated using the kernel method; (**E**) CDI scores significantly differed across the three age groups, consistent with developmental expectations (ANOVA: F = 61.6, *p* = 1.99671e-18). Mean CDI scores were: Group 1 = 0.17 (SD = 0.08), Group 2 = 0.33 (SD = 0.10), and Group 3 = 0.49 (SD = 0.15).

The study was approved by the French National Ethical Committee (OUEST III, approval number 2019-A0337). During the testing session, the infant sat next to one parent in a semi-standardized configuration. The parent was instructed to place their forearm, sleeves rolled up, within the infant’s reach, mimicking the condition of NHP social grooming, where hairy skin serves as a target for tactile interactions (Fig. 1A). To minimize parent-infant interaction, the parent was asked to focus on answering questions from the MacArthur-Bates Communicative Development Inventories: Words and Gestures (CDI-WG)^19,20^, an assessment of the child’s non-verbal and verbal development and from the CDC’s developmental milestones checklist^21^. We hypothesized that the parent’s inattentiveness would increase the infant’s drive for engagement, potentially triggering grooming behaviors. In this context, we anticipated observing a variety of hand actions on the parent’s arm, such as grasping, holding, pinching, and potentially grooming.

Testing sessions occurred in the morning and the afternoon in order to sample parent-infant interactions at various times of the day. Morning (around 10 am) and mid-afternoon (around 4 pm) sessions were most common (Fig. 1B). This variability presented an opportunity to explore whether external daily routines or internal circadian rhythms influenced the likelihood of observing grooming-like behaviors.

Sixty-seven infants participated in the study (*N* = 41, 61.19% in the laboratory; *N* = 26, 38.81% at home; females 38, 56.7%). Investigations were conducted on children aged between 5.5 and 20 months (mean age: 12.0 months), resulting in a total of 108 observations when accounting for longitudinal tracking. The observations were grouped into three categories based on the child’s age at the time of assessment.: Group 1 included infants under 10 months (Mean Age: 8.23 months, SD: 0.88, range 5.5 to 9.5 months), Group 2 included those between 10 and 14 months (Mean Age: 11.43 months, SD: 0.77, range 10 to 13.2 months), and Group 3 included those over 14 months (Mean Age: 15.45 months, SD: 1.64, range 14 to 20 months) (see Fig. 1C and D). In line with their neurodevelopmental stages, the three groups exhibited different performances on the CDI index total score [ANOVA F(2, 105) = 61.6, *p* = 2.00e-18 – eta squared *(η²)* 1.65454/3.0646 = 0.54 (large) – CDI scores: group 1 = 0.17 (0.08), group 2 = 0.33 (0.10), group 3 = 0.49 (0.15)] (see Fig. 1E).

From a qualitative point of view, infants’ grooming behavior typically unfolded as follows: during the session, infants actively sought their parent’s attention through sustained eye contact. While gazing at their parent, infants engaged in a range of manual actions, such as grasping, holding, and pinching, primarily directed at the parent’s forearm. After several unsuccessful attempts to elicit a response, infants shifted their gaze to the arm, by initiating a distinctive motor pattern reminiscent of grooming behaviors typically observed in NHPs. This behavior involved a repetitive sweeping motion of the thumb across the skin, as if searching for something, followed by pinching and lifting movements of the entire hand, resembling the action of removing debris from hair in NHPs (Supplementary Video 1). Interestingly, as in NHPs, some infants maintained visual attention on the area being groomed, adjusting their head position as they searched (Supplementary Video 1). Although grooming behaviors primarily targeted the parent’s arm, they occasionally extended to other body areas, such as the ears, beard, and hair. Interestingly, in some infants, we observed a sophisticated sequence of behaviors strikingly similar to those seen in NHPs: after seemingly removing “impurities” from the parent’s arm, the infant would inspect the retrieved item before bringing it to their mouth. Testing sessions were scheduled at various times throughout the day to accommodate the infants’ needs and the family’s daily routines. As a first step, we developed an ethogram to generate a comprehensive list of infant behaviors observed during the testing sessions. To specifically characterize the distinctive motor features of grooming behavior, our primary analyses focused on a subset of manual behaviors selected for the purposes of this study.

### NHP-like grooming behavior in human infants

**Fig. 2:**
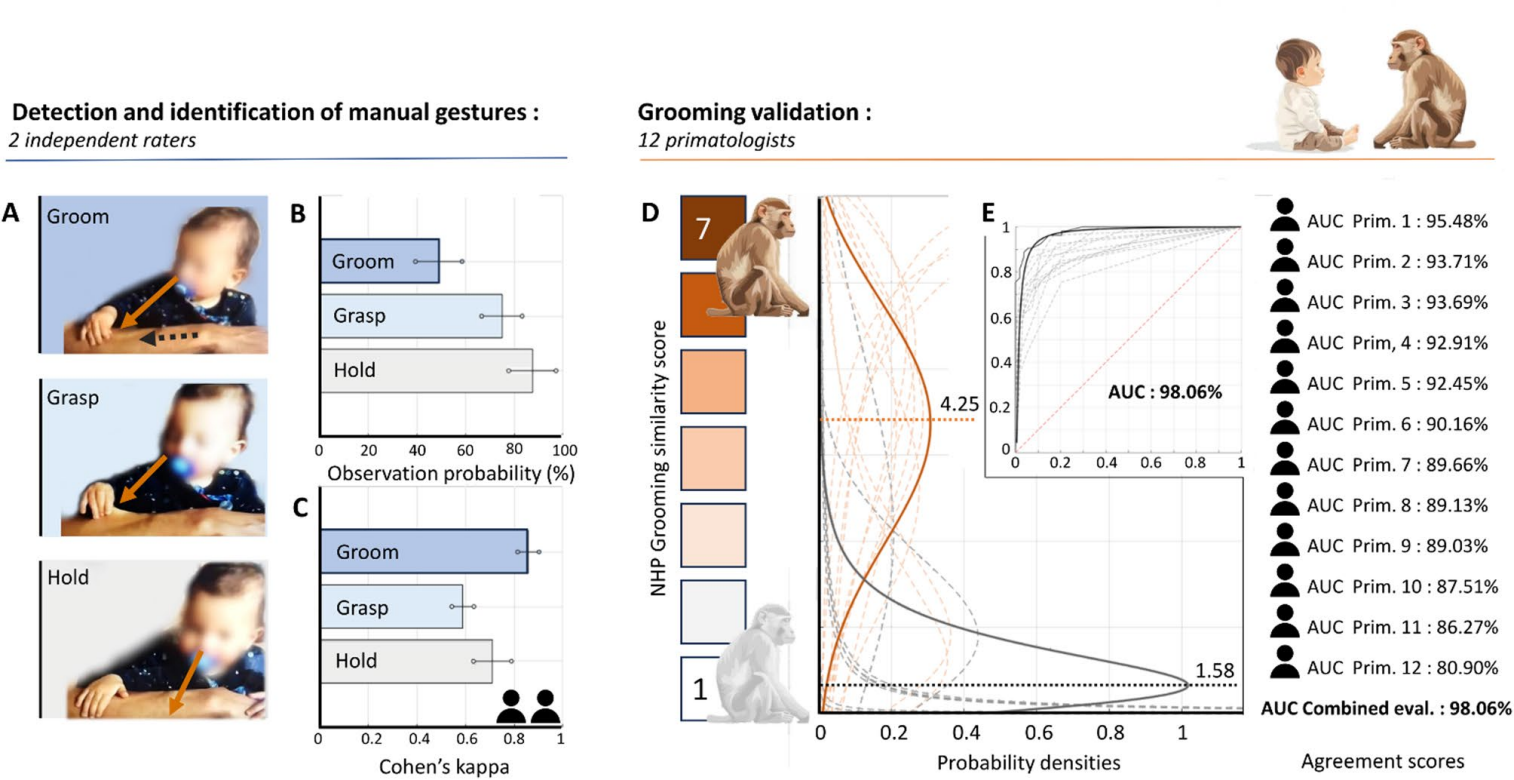
Grooming Behavior Detection and Validation. (**A**) Examples of an infant’s grooming, grasping, and holding behavior identified by two independent evaluators from video recordings. (**B**) The likelihood of observing these behaviors is statistically higher for holding and grasping than for grooming. (**C**) Cohen’s kappa indicates greater agreement between evaluators for grooming than for grasping and holding, suggesting grooming is easier to identify. (**D**) Evaluation of grooming behaviors from 108 video sessions rated by 12 primatologists on a 7-point Likert scale (1: not similar to 7: very similar to NHP grooming). Orange curves represent scores for sessions with grooming, while gray curves represent non-grooming sessions. Primatologists rated grooming behaviors as more similar to NHP grooming than non-grooming sessions; (**E**) Receiver Operating Characteristic (ROC) curves and Area Under the Curve (AUC) were calculated for each primatologist. All rated grooming sessions highly, with AUCs exceeding 80%.

Do human infants exhibit grooming behaviors akin to those of NHP? To address this question, we began by classifying and quantifying the infants’ hand behaviors, focusing on their interactions with the parent’s arm, face, ears, and hair. Two evaluators developed an ethogram to comprehensively describe the infants’ behaviors and independently coded the video recordings from both home and laboratory settings, using the BORIS software^25^. A complete list and definition of these behaviors are provided in Table S1 See Fig. S1 data analysis flowchart and Fig. S2 for an example of BORIS evaluation. The analysis revealed that the most frequently observed behaviors directed at the parent’s body were Holding, Grasping, and Grooming. (Fig. 2A, B). Statistical analysis showed a significant difference in the likelihood of observing each behavior (F(2,261) = 15.35, *p* < 0.001 – eta squared *(η²)* 6.1759 /58.667 = 0.1053 (medium)). Specifically, Holding was observed in 87.5% of sessions (95% CI: 77.80% − 97.20%) and Grasping in 75.00% of sessions (95% CI: 66.70% − 83.30%), both of which occurred more frequently than Grooming, which was observed in 49.07% of sessions (95% CI :39.49% − 58.65%). This suggests that more complex and skillful movements, like grooming, are less likely to be observed than simpler actions such as holding and grasping (Fig. 2B). This pattern implies that grooming may be a more sophisticated motor behavior, requiring specific conditions for its emergence. To ensure the reliability of behavior classification, we assessed the level of agreement between the two evaluators using Cohen’s Kappa (Fig. 2C). Agreement was significantly different among the three behavior (F(2, 173) = 44.03, *p* < 0.001 - eta squared *(η²)* 2.56081/7.5915 = 0.3373 (large)), with Grooming showing the highest inter-rater reliability (k = 0.87; 95% CI: 0.85–0.90), followed by Holding (k = 0.76; 95% CI: 0.71–0.82) and Grasping (k = 0.60; 95% CI: 0.55–0.64). These findings underline the distinctiveness of the grooming action, which was more consistently recognized and identified by both coders compared to the other behaviors.

### Evaluations of grooming behavior by primatologists

The second step of the analysis involved having 12 experienced primatologists assess the similarity between infants’ behavior and NHP grooming behavior. Video recordings were categorized into two groups based on the ethograms developed by the two evaluators: sessions containing grooming behaviors and sessions without grooming behaviors. The primate experts, blind to the evaluators’ classifications, assessed the similarity of the infants’ *gestural and kinematic sequences* to NHP grooming behaviors, with particular attention to the spatiotemporal dynamics of movement using a 7-point Likert scale, where 1 indicates “not at all similar” and 7 indicates “totally similar” (Fig. 2D, Supplementary Analysis 4 for details on methods and analysis). Primatologists’ evaluations differed markedly between sessions labeled as grooming and those without grooming. Across the 107 video‑recorded sessions, mean scores were significantly higher for grooming sessions (4.27 ± 0.96) than for non‑grooming sessions (1.66 ± 0.62), yielding a mean difference of 2.61 ± 0.59 (paired t‑test, *p* = 8.44 × 10⁻⁹). To account for inter‑individual variability in the 7‑point Likert ratings, scores were converted to Z‑scores. The pattern remained robust: grooming sessions received higher standardized ratings (0.69 ± 0.07) than non‑grooming sessions (− 0.68 ± 0.07), with a mean difference of 1.37 ± 0.15 (paired t‑test, *p* = 2.80 × 10⁻¹²). For more details on these analyses see Supplementary Material, Analysis 4. The orange dashed curves in Fig. 2D represent individual experts’ score distributions for grooming-present sessions, while the gray dashed curves illustrate scores for grooming-absent sessions. On average, primatologists assigned significantly higher similarity scores to grooming-present sessions (4.272 ± 1.279) than to grooming-absent sessions (1.657 ± 0.537; t(105) = 13.8276, *p* < 0.001, Cohen d = 2.67 (large)). Receiver Operating Characteristic (ROC) analyses revealed that all 12 primatologists showed strong discrimination between grooming-present and grooming-absent sessions, with Areas Under the Curve (AUC) consistently above 80%. The highest AUC, derived from the average ratings of the 12 primatologists, was 98.06% (Fig. 2E). This demonstrates a clear distinction between sessions with and without grooming, confirming that infants’ grooming-like behaviors resemble those observed in NHPs.

After evaluating the video recordings, primatologists were invited to outline the criteria guiding their similarity judgments. Core features of NHP grooming emerged as guiding principles (see Supplementary Material for a detailed analysis and Tables S2-S8), including specific finger movements such as pinching and plucking, goal-directedness, rhythmicity, and visual focus on the area being groomed. Hand actions were classified as grooming when one or more of these features were present. Behaviors fulfilling these criteria were considered partially homologous to NHP grooming. However, as the primatologists noted, developmental constraints in motor control likely account for the limited proficiency exhibited by human infants. Notably, nearly one-quarter of the observed actions received high similarity scores (23% rated > 5.5 on a 7-point scale), reflecting strong consensus among the 12 experts on the resemblance of these behaviors to NHP grooming.

### Grooming, age and language development

**Fig. 3:**
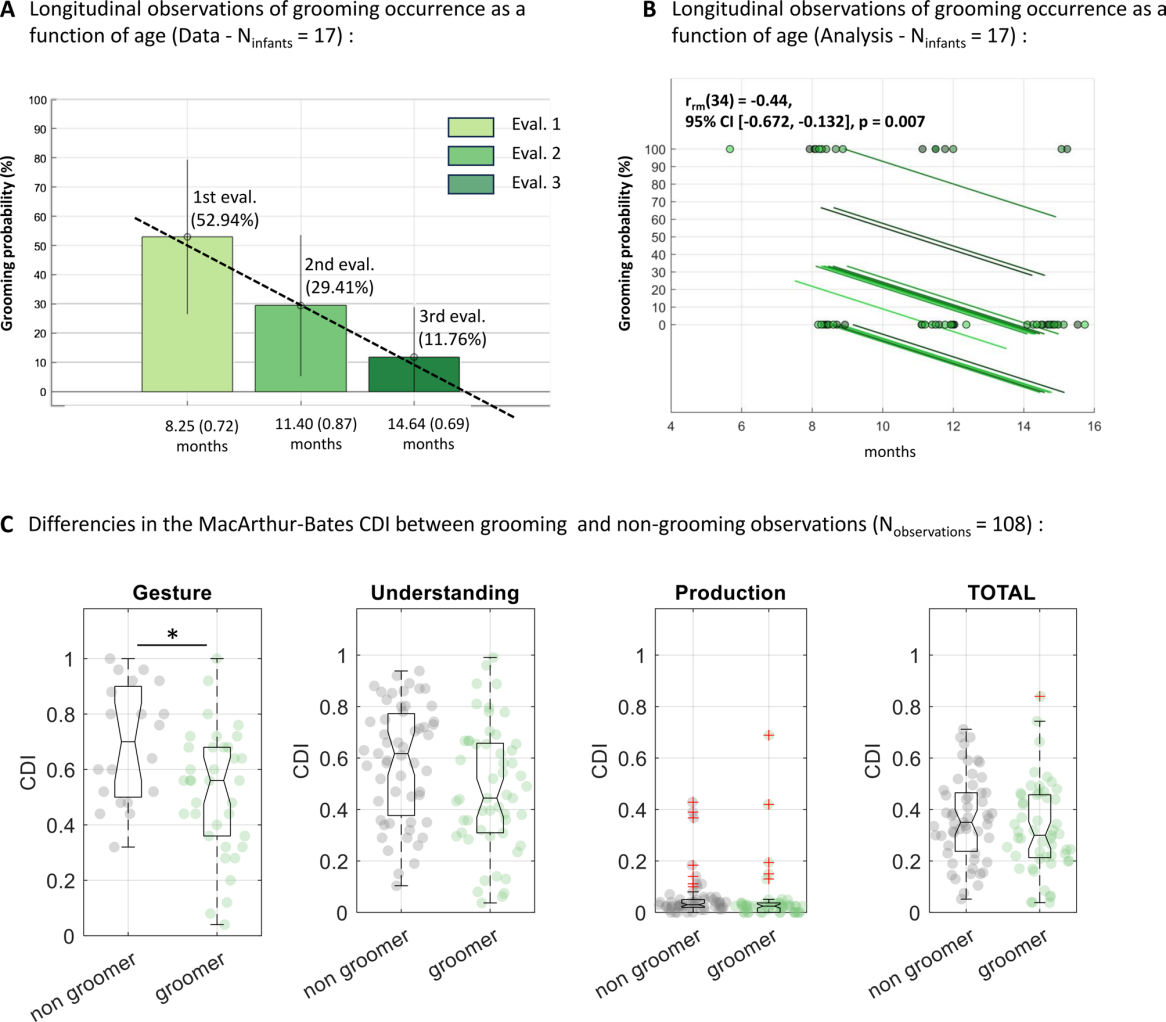
Grooming as a function of age and infants’ communicative development scores. (**A**). Longitudinal measurements from a subgroup of children who were tested at three different ages (*N* = 17). As age increases, the likelihood of observing grooming behavior decreases. (**B**) This trend is confirmed by a repeated measures correlation analysis (r_rm_(34) = -0.44, 95% CI [-0.672, -0.132], *p* = 0.007) and compared to grasping behaviors, it shows that is specific to grooming (Supplementary Analysis 5). (**C**). During the observation period, most children are preverbal. Therefore, most of the development of communication skills is assessed by the Gesture and Understanding items. Groomers scored significantly lower on the CDI Gesture subscale compared to non-groomers (*p* = 0.0067, *p* < 0.05, Bonferroni corrected for multiple comparisons).

We investigated the relationship between age, language development, and grooming behavior to determine if grooming is a transient motor behavior that fades as language skills develop. Across all observation sessions, there was no significant correlation between the infants’ age and the likelihood of observing grooming behavior [r(107) = − 0.18, *p* = 0.067]. To gain deeper insights, we conducted a longitudinal study with 17 infants, tested at three different ages. The results revealed a decline in grooming behavior with age (Session 1 : 8.25 ± 0.72 months: 52.94%; Session 2: 11.40 ± 0.87 months: 29.41%; Session 3: 14.64 ± 0.69 months: 11.76%). Repeated measure correlation confirmed this trend (r_rm_[34]= -0.44, 95% C.I.: [-0.672, -0.132], *p* = 0.007, Fig. 3A). These findings suggest that grooming behavior in infants declines between the ages of 8 and 15 months.

Could grooming behaviors in human infants be linked to the development of their communication skills? To explore this, we assessed infants’ language development using the MacArthur-Bates Communicative Development Inventories (CDI), a parent-reported measure of early communication abilities, including gesture use and word production (see Methods). Infants who exhibited grooming behavior scored significantly lower on the CDI gesture subscale (M = 0.5189, SD = 0.2248) compared to those who did not exhibit grooming behavior (M = 0.6920, SD = 0.2117; t(54)=-2.818, *p* = 0.0067, Cohen’s d = 0.16 (small)) with significance maintained after Bonferroni correction (Fig. 3B). This suggest that the decline in grooming behavior occurs during an optimal temporal window when infants are acquiring new communication gestures (CDI Gesture M = 0.58, SD = 0.23) and before the onset of verbal language production (CDI Production M = 0.05, SD = 0.1). See Fig. 3C for differences for groomers and non-groomers in CDI scores. See also Supplementary for more analyses, Fig. S8-12 and Tables S2-14).

### Contextual and temporal modulation of grooming behavior

**Fig. 4:**
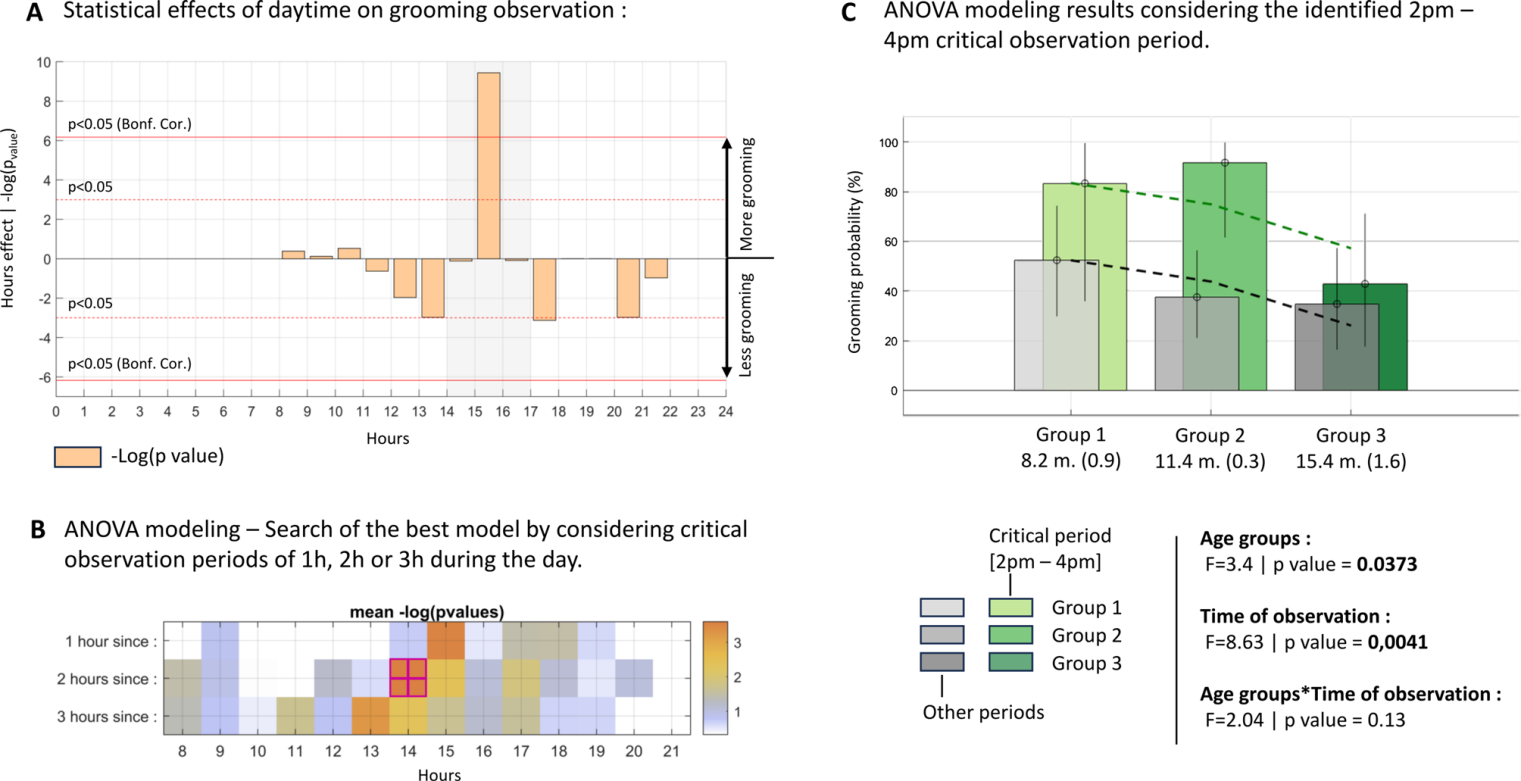
Probability of observing grooming behavior varies with time of observation. (**A**). The probability of observing a grooming behavior was significantly higher between 3 pm and 4 pm; (**B**). An ANOVA model incorporating age, time of day, and their interaction indicated that the optimal observation period is between 2 PM and 4 PM. (**C**). Grooming probabilities for the three age groups observed between 2 PM and 4 PM are shown in green, along with their 95% confidence intervals. Grooming probabilities for the same age groups observed outside this peak period are presented in grey, also with their 95% confidence intervals. The dashed lines indicate the marginal mean values for grooming, not considering the non-significant interaction between age group and time of observation. These results corroborate the age-related decline in grooming behavior and delineate an early-afternoon period as optimal for observation. Alternative models that consider the other potential confounding variables in the model yielded the same result (see Supplementary Analysis 7).

Could specific contexts trigger or enhance grooming behaviors? To investigate this, we examined the relationship between grooming behavior and contextual factors, including the testing environment (laboratory vs. home), the infant’s gender, and the time of the day. No significant differences were found in grooming probability between laboratory and home environments (Home 41.7%, Lab 58.3%; Chi2 = 2.9640, *p* = 0.0851), or between boys and girls (Girls 51.7%, Boys 46%; Chi2 = 0.3520, *p* = 0.5530). However, the analysis of daily observation times revealed a striking temporal pattern. The independence of the hour of observation on the probability of detecting a grooming behavior was evaluated using a contingency table and Chi2 tests with the family-wise error rate (FWER) controlled for multiple comparisons using the Bonferroni correction. The probability of observing a grooming behavior was significantly higher between 3pm and 4pm (76%) compared to the overall observation period (49.07%; Chi2 = 9.44, *p* < 0.001, Bonferroni corrected *p* < 0.05) (Fig. 4A). This observation aligns with the circadian rhythm of beta-endorphin levels, which reach their lowest point (bathyphase) between 2 pm and 5 pm (Fig. 4B, purple curve), as estimated from experimental data in newborns and in adults^22,23^(purple dots). By contrast, no time-of-day effect was observed for grasping or holding behaviors (Supplementary Fig. 4), suggesting that the afternoon increase in grooming activity does not reflect a circadian influence on general motor activity. Previous studies have causally linked beta-endorphin activity to the likelihood of grooming in NHPs^6,24^, and recent research in humans has connected this rhythm to changes in pain perception^26^. Our findings suggest that this temporal window may represent a particularly favorable period for observing grooming behavior in infants, potentially reflecting a shared neurophysiological mechanism, although this interpretation remains speculative at this stage.

To better understand how age and time of day influence the likelihood of observing grooming behavior, we conducted a two-way ANOVA. We tested multiple observation windows between 8 AM and 10 PM. Our analysis revealed that grooming behavior is most likely to be observed in younger infants during an optimal two-hour window from 2 PM to 4 PM. Both age groups (F(2,102) = 3.4, *p* = 0.0373 - eta squared *(η²)* 1.5404/26.9907 = 0.0571 (small)) and observation time (F(1,102) = 8.63, *p* = 0.0041 - eta squared *(η²)* 1.9571/26.9907 = 0.0725 (medium)) were significant factors. However, there was no significant interaction between age group and observation time (F(2,102) = 2.04, *p* = 0.13)), suggesting that age and time of day exert independent effects on grooming likelihood. Post-hoc comparisons revealed no age range in which the effect of age reached statistical significance. Figure Fig. 4C illustrates grooming probabilities for the three age groups between 2 PM and 4 PM (green bars), alongside their 95% confidence intervals. For comparison, grooming probabilities for the same age groups observed outside this optimal temporal window are shown in grey, also with their 95% confidence intervals. Post-hoc analyses did not identify a distinct age period driving the observed decrease in grooming frequency. Dashed lines represent the marginal mean probabilities of grooming, reflecting the independent effects of age and time of observation. These results suggest that younger infants are more likely to display grooming behavior during the early afternoon, aligning with the bathyphase of the beta-endorphin circadian rhythm.

## Discussion

This study reveals, for the first time, that human infants engage in grooming-like behaviors that closely resemble grooming observed in NHPs. Primatologists provided similarity scores for each behavior, with several grooming behaviors receiving high scores, reflecting a strong consensus that a category of infants’ behaviors exhibit key characteristics of NHP grooming. The frequency of grooming was found to decrease with age and the development of language skills, as shown in longitudinal observations from 8 to 15 months. Our findings further reveal that the probability of observing grooming was significantly higher between 2 and 4 PM, coinciding with the bathyphase of the circadian rhythm of beta-endorphins, a neurochemical known to modulate pain and stress^27–30^, but also some social behaviors^6,31,32^.

To date, no studies have directly linked circadian variations in β-endorphin levels with grooming behavior. However, a study in non-human primate (Talapoin monkeys) has shown that grooming activates C-tactile afferents, which in turn trigger the release of β-endorphins^3^. This demonstrate that grooming interactions significantly affect brain opioid systems, particularly β-endorphin activity. Notably, blockade of opioid receptors increased the motivation to be groomed, suggesting that reduced opioid signaling enhances grooming-seeking behavior. Conversely, administration of morphine (an opioid agonist) decreased the motivation to be groomed. Together, these findings support the idea that β-endorphins are key mediators of social attachment and may have played an important role in the evolution of primate sociality^3,11^. Additionally, there is evidence that β-endorphin levels in non-human primates follow a circadian rhythm, although again this has not yet been directly linked to grooming behavior^11^.

A central question is whether infant grooming reflects exploratory motor activity, so that similar behaviors would occur with strangers or non-social targets^33^, or whether it represents a socially motivated attempt to connect with familiar caregivers, as seen in other primates^34^. To test this, a subset of infants was observed in two control conditions: interaction with a fur collar and interaction with a human stranger (Supplementary Analysis 8, Table S15). Infants did not engage in grooming in either control condition, supporting the interpretation that grooming in infancy is context-specific and functions as a behavior directed toward close social partners, rather than as a general exploratory action.

### Similarity between infant and NHP grooming

To analyze infant grooming behavior, video sequences were recorded and evaluated by two independent coders, followed by assessments by 12 experts, including neuroscientists and ethologists with extensive familiarity with NHP behavior. These experts rated the similarity of the behavior to NHP grooming. Grooming behavior exhibited a distinct motor profile, characterized by thumb–index movements involving sweeping, pinching, and plucking, which enabled experts to distinguish them from general manual actions such as holding and grasping. The primatologists’ evaluations highlighted that infant grooming shared several key features of NHP grooming, such as goal-directed action, bilateral hand coordination, gaze fixation on the groomed area, and rhythmic repetition of movements. These features were demonstrated at a level of proficiency consistent with the infants’ stage of motor development.

### Functional role of grooming in infants: Bonding, Communication, or exploration?

The functional role of grooming-like behavior in infants remains an open question. In NHPs, grooming serves as a social bonding mechanism, reinforcing social ties and reducing stress^35–37^. A similar function may exist in human infants, as grooming-like behaviors were most frequently observed when the caregiver was unresponsive, a context that aligns with attachment theory, in which infants seek proximity and attention from a caregiver.^38^[As a reminder, all our participants were middle-class, and patterns of attachment vary considerably across cultural settings (see e.g., ^39^).] Grooming may thus serve as a tactile form of social engagement in the absence of verbal communication. This developmental pattern may lend support to Dunbar’s hypothesis^1^that language emerged as a form of vocal grooming, enabling individuals to maintain social bonds more efficiently as group sizes increased; the corresponding decline in physical grooming may indicate a functional shift from tactile to verbal modes of affiliation. Alternatively, grooming-like behavior may be part of a broader repertoire of exploratory actions performed by infants on accessible body surfaces. From this perspective, grooming behavior could be exploratory in nature, with tactile feedback from the skin reinforcing these repetitive behaviors. However, the repetitive, rhythmic nature of grooming-like behavior and its strong association with caregiver interaction suggest a more specific role in attachment or bonding. At this stage, each of these hypotheses requires additional in-depth investigation to thoroughly evaluate their validity and potential implications.

### Diurnal variation in grooming: links to Beta-Endorphin rhythms

One of the most striking findings was the increase in grooming behavior observed between 2 and 4 PM. This pattern aligns with the bathyphase (low point) of the circadian beta-endorphin rhythm as measured in neonates and which has been implicated in stress regulation^22,23^. In NHPs, beta-endorphin is strongly linked to social grooming: beta-endorphin concentrations are influenced by grooming relationships^6^and opiate antagonists stimulate this affiliative behavior^24^The present data do not allow us to determine whether circadian processes causally influence grooming or social bonding behavior. However, the observed temporal pattern is consistent with the possibility that fluctuations in internal physiological state across the circadian cycle, including, but not limited to, endorphin-related processes, may be associated with variation in the expression of grooming behavior. This interpretation is necessarily speculative and meant to generate hypotheses for future work rather than to provide direct evidence of a causal link.

If future studies confirm this, it would suggest that social bonding behaviors in human infants are modulated by physiological rhythms simultaneously with other circadian physiological regulations such as pain perception^26^.

### Evolutionary implications: persistence vs. Extinction of latent motor programs

The observation of NHP grooming-like behavior in human infants offers a rare opportunity to investigate the persistence of ancestral motor patterns within the human behavioral repertoire. Evolutionary psychology has long debated whether behaviors that are no longer expressed in adulthood are entirely extinguished or remain latent, reemerging under certain conditions^1^. Our findings support the latter interpretation, indicating that the neural circuitry underlying grooming behavior remains accessible during early development, even if it is no longer prominently expressed in adult human social interactions.

We further propose that grooming-like behavior may constitute a vestigial trait, transiently expressed in early childhood before being supplanted by more sophisticated forms of communication. Notably, the longitudinal analysis revealed that grooming behaviors began to decline around eight months of age and had disappeared by approximately fifteen months. This decline coincides with the emergence of communicative gestures, as measured by MacArthur-Bates inventory scores. Such developmental trajectory is reminiscent of other vestigial motor behaviors, such as the neonatal stepping reflex, which disappears as infants develop more refined locomotor control^40^. Unlike simple motor responses, grooming is a goal-directed behavior. Its transient expression during early development may nevertheless arise from the reactivation of evolutionarily conserved motor pathways that are accessible only within a restricted developmental window. We hypothesize that this early expression functions as a transitional mechanism, providing neural and behavioral support that promotes the developement of socially oriented behaviors. In this view, grooming may represent an ancestral motor program repurposed during ontogeny to support the initiation of social contact. This developmental pattern may lend support to Dunbar’s hypothesis^1^that language emerged as a form of verbal grooming, enabling individuals to maintain social contact more efficiently as group sizes increased; the corresponding decline in physical grooming may indicate a functional shift from tactile to language modes of affiliation.

The gestural behaviors observed in children in our study may reflect motor programs that are also engaged in other goal-directed actions. Yet, the remarkable resemblance between the motor sequences we documented and the grooming patterns of non-human primates (NHPs) suggests that, although NHP-like grooming no longer serves a functional purpose in adult humans, the underlying motor program persists as an ancestral remnant of our evolutionary past. This aligns with other motor behaviors of high ethological significance, such as hand-to-mouth movements^41^. The transient expression of this grooming behavior during a specific developmental window, as observed here, further supports the notion that it represents an evolutionarily conserved vestige, shared across species but gradually suppressed in adult humans, at least in the form seen in NHP groups.

Although certain caregiving behaviors performed by adults (such as wiping, washing, brushing, combing, or cleaning) can be seen as continuations of grooming into adulthood^42^, human social bonding primarily depends on conversation, storytelling, shared meals, rituals, and cooperative work rather than reciprocal fur-picking or parasite removal. In other words, while adults still engage in behaviors reminiscent of grooming, these actions have been reshaped by cultural and social norms and no longer retain the specific motor patterns characteristic of non-human primate grooming. The novel contribution of the present study is the demonstration that infants exhibit the ancient form of grooming whose manual structure, action sequencing, and sensorimotor organization closely parallels that of NHP grooming, a pattern not captured by general descriptions of cleaning or caretaking. We propose that this NHP-like grooming in infancy represents an ancestral form of social bonding, expressed when other modalities are not yet available at this developmental stage. Our results show that this specific grooming pattern emerges and disappears within a well-defined developmental window and is unlikely to persist into adulthood.

We acknowledge ethnographic reports documenting frequent tactile caregiving in some non‑WEIRD human groups (e.g., caregiver-to-child grooming practices described by^42^). Such behaviors can be formally similar to NHP grooming in their tactile form, but they often differ in social function and structure^6,10^. Unlike the time‑intensive, reciprocal grooming networks that structure many primate societies, human tactile caregiving is frequently episodic or instrumental and is complemented or largely supplanted by language, shared activities, and culturally organized rituals for maintaining social bonds. Consequently, we interpret the grooming‑like behaviors observed in humans as transient, evolutionarily retained analogues rather than direct functional equivalents of primate alliance‑forming grooming networks^17,16,18^.

^14,15^ Our study included infants from both Chinese and European populations, all of whom exhibited grooming-like behaviors, suggesting that these behaviors reflect a culturally independent developmental expression of an ancestral behavioral repertoire. Thus, the observed grooming-like behaviors in infants may represent a developmental window during which this conserved actions are more readily expressed, before it is overshadowed by more efficient forms of social bonding, such as symbolic behaviors and language.

### Conclusion and future perspectives

While this study provides robust support for key findings, a few methodological considerations warrant attention. First, the testing environment (home vs. laboratory) may have influenced grooming frequency. Parents reported that some infants who did not display grooming during lab sessions later exhibited it when similar conditions were recreated at home, suggesting that grooming frequency may have been underestimated in the lab. Second, the modest sample size for longitudinal tracking could limit the generalizability of findings regarding the transient nature of grooming, although our results were statistically robust. Finally, while the observed peak in grooming between 2 and 4 PM aligns with the bathyphase of the beta-endorphin cycle reported in the literature, direct measurement of beta-endorphin levels in infants and multiple assessments of the same infant at different times of the day could provide further evidence for this link. Despite these considerations, the study’s consistent findings across age groups, times of day, and testing environments offer compelling evidence for the transient expression of NHP-like grooming behaviors in human infants.

In conclusion, this study reveals that NHP-like grooming behaviors are not uncommon in human infants, occurring spontaneously during interactions with caregivers. This finding challenges the assumption that grooming has been entirely replaced by verbal communication in humans. Instead, it suggests that grooming persists as a latent motor program, emerging in early childhood as a potential tool for attention-seeking, relationship maintenance, or stress regulation. These findings also support the broader idea that evolution has preserved elements of social grooming in the human motor repertoire, at least during a critical developmental period marked by emerging fine motor control and preceding the acquisition of verbal communication. Future research could further explore this latent motor program, including its links with attachment, tactile bonding, and early emotional development. Such investigations could shed light on the role of grooming-like behaviors in shaping early social interactions and the broader developmental trajectory of human social cognition.

## Methods

### Experimental procedure

The protocol consisted of 108 sessions of observations of parent-infant interactions, recorded in a controlled environment in the laboratory or in a more ecological environment at home. The parent was sitting close to the child, and exposed her/his arm in front of him, while the experimenter was asking questions to fill out a questionnaire [i.e., Child Development Inventory (CDI)^19,43^. Also, we asked the parent to roll up their sleeves, remove any jewelry, expose their arm, and place it within reach of the child. We recorded the entire session for offline coding analysis. Observations were made between 8am and 10pm, depending on the availability of families. During the interview, parents were instructed not to respond to their child’s solicitations throughout the testing session, while completing the 10-minute questionnaire.

In total, we collected a total of 108 observations: 48 observations (*N* = 41 infants) in the lab and 60 observations (*N* = 26 infants) at home via video calls. This means that certain infants have been observed longitudinally (*N* = 17 infants with at least 3 observations) with an interval of about 3 months between each observation. The study was approved by the Ethical Committee (CPP, OUEST III 2019-A0337). Research was performed in accordance with the Declaration of Helsinki. Written informed consent to participate in the study and signed permission for video recording and dissemination were obtained from parents before participation. Informed consent was signed from legal guardians for publication of identifying information/images in an online open-access publication.

#### Home video-call observation

The observation was conducted via video call. The experimenter interacted with the parents and infants through the camera and the interaction was video-recorded for offline analysis. Before the meeting, parents completed the online consent form and received information about the study. During the videotaped interview, the infant was accompanied by only one parent who was instructed to keep her/his bare arm on the table in front of the infant. The parent was instructed to ignore the infant as much as possible and answer the experimenter’s questions about the infant’s motor, and verbal development.

#### Laboratory observation

The experimental setup was similar to the video call observation instead of taking place in the laboratory room (Fig. 1A). The infant was accommodated in a high chair. A single parent remained near the infant and was instructed to hold the forearm resting on the high chair food tray within reach of the infant. Parent-infant interactions were videotaped throughout the visit with a Sony HDR-CX405 video camera for offline analysis.

The observation length varied across infants with a mean duration of 10.60 min (± 3.71 SD, range: 6.10 to 24.58 min). During the testing, the experimenter administered the parent the Checklist of Developmental Milestones and the MacArthur-Bates Communicative Development Inventories: Words and Gestures (CDI-W;^19–21^).

#### Studied population

A total of 67 French and Chinese healthy infants, aged between 6 and 20 months (Mean 12 months - S.D. 3 months) were recruited for the study (*N* = 38 females, 56.7%). French infants (*N* = 47) were recruited by the Baby Lab at the CNRS Institut des Sciences Cognitives for either in laboratory or home video-call observations. Chinese infants (*N* = 20 living in China) were recruited via online flyers for home video-call observations only. A selected group of infants were tested longitudinally (*N* = 17). Basic socioeconomic data were collected, such as parents’ employment status: all families belonged to the middle class and both parents were working. Future studies might collect socioeconomic data to investigate their possible role in infant care and the link with grooming behavior.

#### Checklist of developmental milestones

To ensure that all infants reached their developmental age, parents completed a developmental milestone questionnaire from the *Centers for Disease Control and Prevention*^21^. The milestone checklist includes a set of activities that infants should be able to perform at a specific age. It includes four areas: social/emotional, language/communication, cognitive, and movement/physical. For this evaluation, the questionnaire versions are adapted to the child’s age. (9, 12, or 18 months).

#### Language development assessment

The MacArthur-Bates Communicative Development Inventories: Words and Gestures (CDI-WG ^19,20,44^) was administered to parents, to assess the infant’s language development. The CDIs were used to obtain the developmental trajectory of infants’ language abilities. Two distinct versions of the CDI-WG were administered according to the infant’s age (12 months or 18 months). The CDI-WG 12-months version included: (i) Gesture production (25-items), (ii) Vocabulary comprehension (81-words) and (iii) Vocabulary production (81-words). The CDI-WG 18-months version included: (i) Gesture production (25-item), (ii) Vocabulary comprehension (98-words) and (iii) Vocabulary production (98-words). Each sub-score is rated on a scale from 0 to 1, calculated as the sum of expressed behaviors divided by the total number of behaviors considered. The total score is the average of these sub-scores, also ranging from 0 to 1.

The effect of age on CDI was tested on the three age groups that constitute our population using 95% confidence intervals, ANOVA, and post-hoc comparisons with Tukey’s HSD.

### Coding and analysis of observations

#### Behavior detection

Behavioral coding analysis was performed by two independent experimental psychologists on the videos recorded for each child using the BORIS software^25^. We first defined an Ethogram, which is a list of selected behaviors that infants could exhibit over their parent (i.e., holding, grasping, and grooming) during the testing (See Table S1 for details of observed behaviors). The motor actions of both hands were coded separately, frame by frame, and combined in the final dataset.

The first behavior of interest was a grooming action resembling that observed in NHPs. This behavior was identified when the child used a precision pinch grip, typically involving the thumb and one or more fingers, to manipulate the parent’s skin or body hair. The action was characterized by longitudinal movements along the body surface, often including sweeping and plucking behavior. For a grooming event to be confirmed, both coders had to independently detect the behavior occurring simultaneously. Two other behaviors involving the infant’s hands that could be confused with grooming were also considered: A holding behavior, where the child uses his hands on the parent harm to maintain his position and an active grasping behavior in which the child grasps the parent’s skin while looking at the area being manipulated.

Agreement between the two coders on detecting the motor behavior was assessed using Cohen’s kappa coefficient (κ), where: <0.20 = poor agreement; 0.21–0.40 = fair agreement; 0.41–0.60 = moderate agreement; 0.61–0.80 = good agreement; and > 0.80 = very good agreement. A Cohen’s kappa coefficient was calculated for each behavior and each of the 108 observations/recording sessions. The two variables studied are the probability of observing the behavior and the mean value of the kappa coefficient associated with this behavior. Effects are compared using 95% confidence intervals, ANOVAs and post-hoc comparisons with the Tukey’s honest significance test (Tukey’s HSD).

Our study assessed grooming through the lens of hand and finger movement patterns, with a specific focus on precision grip, to enable comparisons with the same motor behavior in our sample of children. However, we acknowledge that grooming in chimpanzees encompasses a broader range of manual actions. For example, it can involve the use of single digits for scratching or poking^1,45^, sweeping motions performed with the back of an open hand^46^, as well as precision grips for more targeted removals^47^.

#### Behavior validation

A total of 107 hand movement sequences of Home video calls (*N* = 59) and Laboratory observations (*N* = 48) from the initial behavioral coding were submitted to twelve primatologists who had extensive experience in observing NHP grooming behavior (one observation was not evaluated by the primatologist because no grooming / grasping or holding movement was present in the recorded video). We presented to the primatologists only the video clips showing hand movements that had been previously detected and independently evaluated by both coders. Hand movements were identified in 107 of the 108 recorded sessions; therefore, one session was not evaluated by the primatologists, as it contained no hand movements to assess. Each primatologist watched the video sequences, played one by one in a random order, rated to which extent each selected hand movement, resembled the typical NHP grooming behavior, on a Likert scale (from 1 = not at all similar to NHP grooming to 7 = very similar to NHP grooming). Using this procedure, 12 NHP similarity scores were calculated for each observation session (See Tables S2-S8 for primatologists’ evaluation details).

First, we calculated the mean rating score for sessions with and without grooming as assessed by the two initial coders and a t-test was performed to check if primatologists scored differently ‘grooming’ and ‘non-grooming’ sessions. As each primatologist may have a different strategy for assessing behavior on the Likert scale, a second, more robust analysis with regard to inter-individual differences was implemented by exploring, the distributions of scores attributed for each observation session. Based on these distributions, Receiver Operating Characteristic (ROC) curves and the corresponding Area Under the Curve (AUC) can be calculated to measure how distinct each primatologist’s evaluation was between sessions where a grooming action had been detected and those where it had not. The closer the ROC curve is to the upper left corner of the graph, the higher the validation of the initial classification performed by the two initial coders. The ideal ROC curve thus has an AUC = 1.0 or 100%, the evaluation made by the primatologist can be used to classify grooming present and grooming absent recording sessions with a sensitivity = 1 and a false positive rate = 0 (specificity = 1).

#### Non-neurodevelopmental factors influencing the probability of observing grooming behavior

The independence of observation hour, sex, and context/environment (home vs. laboratory) on the probability of detecting grooming behavior was evaluated using contingency tables and Chi-square tests. Multiple comparisons were controlled by adjusting the family-wise error rate (FWER) with the Bonferroni correction. Contingency tables were constructed as 2 (Grooming / No Grooming) × N, with *N* = 2 for sex and context/environment, and *N* = 14 for observation hours (8 a.m. to 9 p.m.). To illustrate and discuss the potential influence of observation hour, we represented the theoretical circadian rhythm of beta-endorphin, estimated from experimental data^22,23^, using a simple 24-hour sinusoidal fit (See also Fig. S3-S7 on probability of observing grooming behavior).

#### Neurodevelopmental factors influencing the probability of observing grooming behavior

The relationship between the probability of observing grooming behavior and age was tested first across all observation session (*N* = 108) using simple Pearson correlation, then across subject tested longitudinally (*N* = 17) using repeated measure correlation (rmcorr^48^with the rmcorrShiny application^49^.

To study the relationship between the probability of observing grooming behavior and neurodevelopmental level, we performed two samples t-test on CDI scores between groomers and non-groomers that were not included in the original manuscript due to the limited number of infants tested,

Considering that both age groups and time of day influence the probability of observing grooming behavior, to best model the influence of these two factors we searched the two-way ANOVA model with interaction that best fit the observed data, testing all one, two- or three-hours periods between 8 am and 10 pm. The criterion for model selection was the minimization of the sum of p-values for hours, age, and age x hours interaction effects.

## Supplementary Information

Below is the link to the electronic supplementary material.

Supplementary Material 1

Supplementary Material 2

## Data Availability

The dataset generated and analyzed in the current study is available on the Open Science Framework repository at https://osf.io/vt3ya/. Infants’ video recordings (home/laboratory) are available upon request (see Supplementary video 1 as an example).
