# Supplementary material for: Revisiting our primate roots in infants grooming
Source: Sci Rep. 2026 Feb 13;16:8783. doi: 10.1038/s41598-026-39909-2 (PMC12982471; doi:10.1038/s41598-026-39909-2)
Supplement: Supplementary file 2 — Supplementary Material 2 [file 41598_2026_39909_MOESM2_ESM.pdf]

Supplementary Material

Mai et al.,

Supplementary Video 1. Example of video recording.

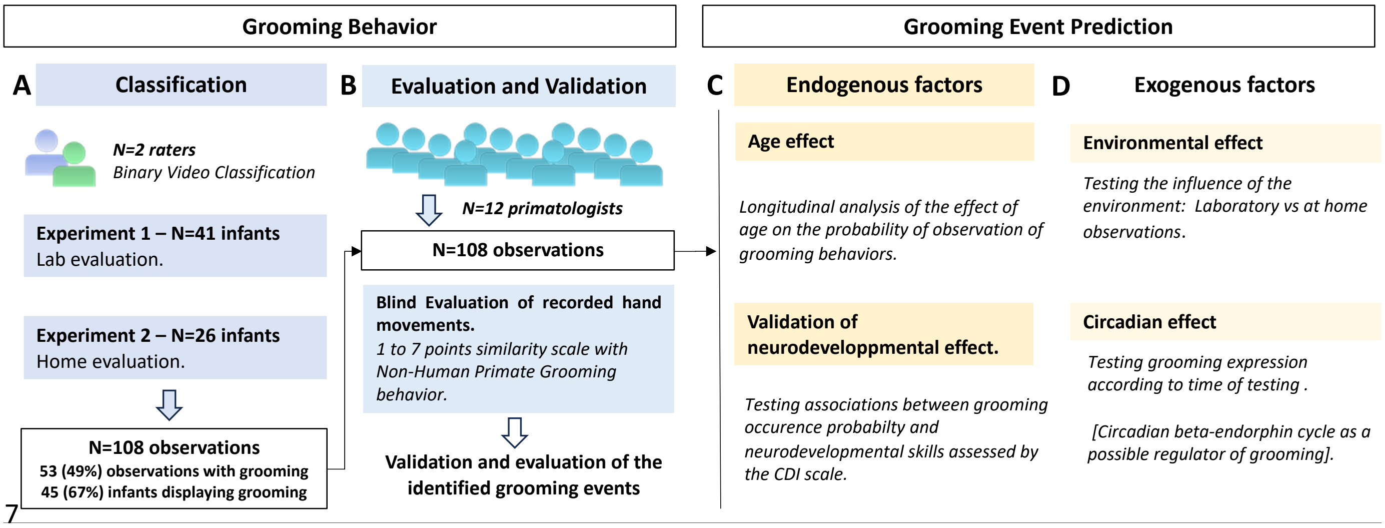

Supplementary Figure 1. Data analysis flowchart.

Supplementary Table 1: Behavioral Observation Research Interactive Software Ethogram

Behavior category	Behavior code	Description
Behavior of interest	**Grooming**	The 'finger-and-thumb' precision grip on the body surface (head, chin, arm, back, etc.) of the parent alternating with sweeping, poking, pinching gestures involving selected fingers or whole hand.
Behavior of interest	**Grasping**	Using the fingers/Hands to scratch parent's body surface or clothes.
Behavior of interest	**Holding**	Using the hand or palm to hold the parent.
Observed behavior	Taping	Using the hand or palm to tap on parent's body surface or clothes.
Observed behavior	Look at	The infants directly gaze at the parent's face
Observed behavior	Vocalization	Phonic production without semantic meaning (e.g., Ah, Oh) or word-like phonic production (e.g., baba, mama).
Observed behavior	Mouth	Use of mouth to suck/lick the objects (other than pacifier), parent's body (e.g., finger, hand, arm and hair), own body (e.g. hand, finger).
Observed behavior	Upset	The eyelids tense, the inner eyebrows descend,<br>and the lips are pressed together to express a feeling of annoyance or displeasure.
Observed behavior	Cry	Release of tears, frequently accompanied by cries and sobs.
Observed behavior	Smile	The corners of the mouth stretch upwards and outwards as an obvious smiling expression.<br>Or the corners of the mouth drawn backwards and upwards, separating the lips, such that the upper and lower teeth are exposed as laughing expression.
Observed behavior	Self-touching	The baby is using hand or finger to touch/grasp/scrach own body part or clothes.
Observed behavior	Sit	The baby is sitting on the baby seat, not standing up.
Observed behavior	Hug	The baby not sitting on the baby seat, standing up and mother hugging him/her.
Observed behavior	Stand	The baby standing up on the baby seat or on mother's body.

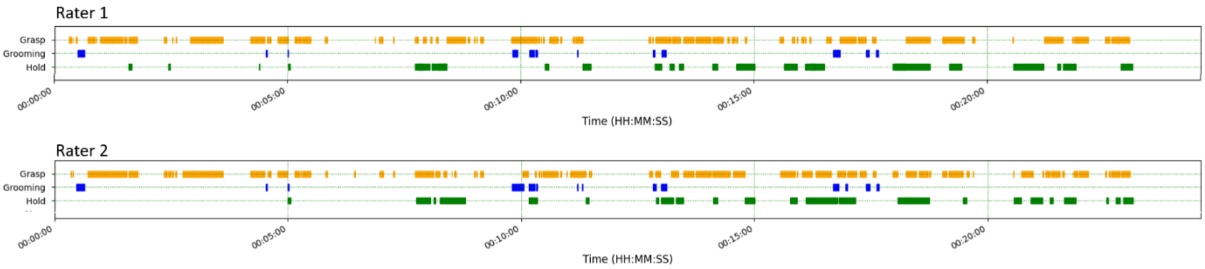

Supplementary Figure 2: Example of BORIS (*Behavioral Observation Research Interactive Software*) evaluation.

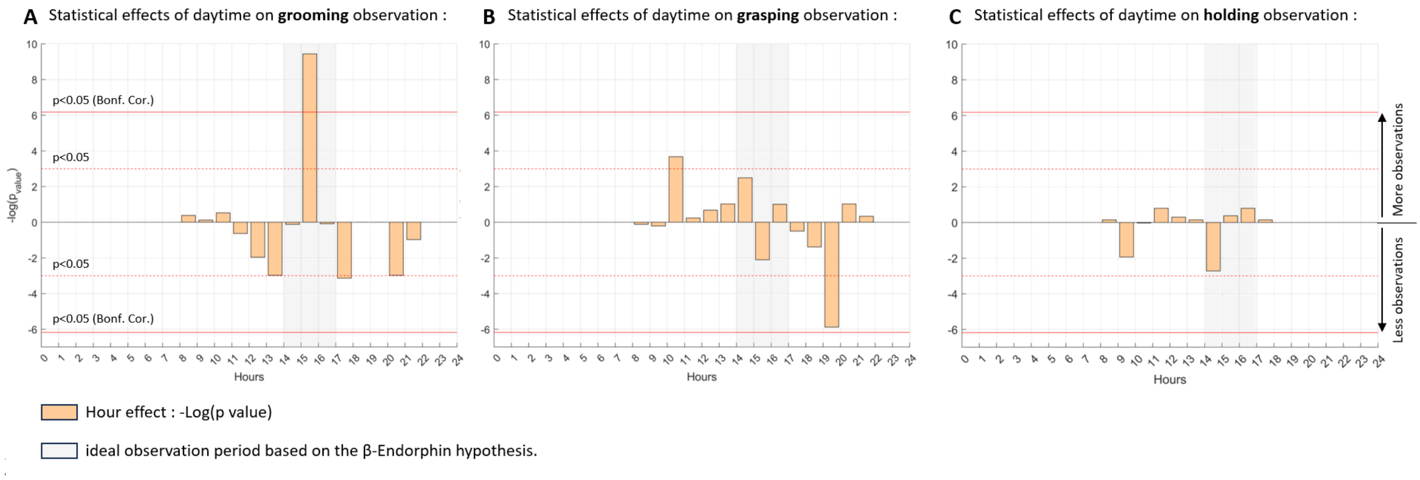

Supplementary Figure 3: Hour effect Same analysis as in Figure 4 (see main text). In addition to the probability of observing Grooming (**A**), we show the probabilities of observing Grasping (**B**) and Holding (**C**). Among these behaviors, only Grooming exhibits a distinct temporal window in which the probability of occurrence is statistically different from that observed in other windows.

Supplementary Analyses

The supplementary analyses 1,2, 3 and 4 are available at: https://osf.io/vt3ya/ OSF project – Grooming_SuppAnalysis.m matlab code.

The supplementary analyses 5, 5b, 6 and 7 are available at: https://osf.io/vt3ya/ OSF project – Grooming_AnalysisV2.m revised matlab code.

Supplementary Analysis 1: Grooming events statistics

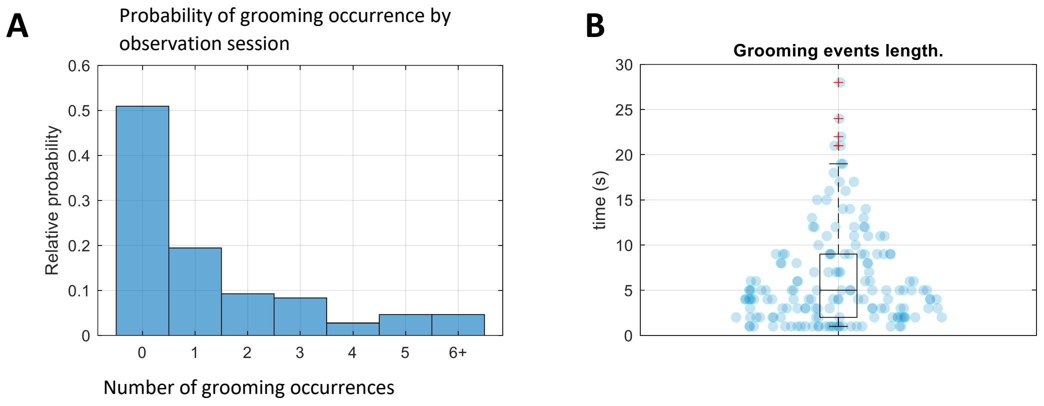

Supplementary Figure 4. Grooming events statistics **(A)** Distribution of the number of grooming occurrences reported per observation session of our dataset. Among the 128 observation sessions, if a grooming event was observed in a session, the median number of grooming events observed by session is 2 (mean 3.19 s.d. 4). **(B)** Swarmplot showing the distribution of the grooming events length for the 169 grooming events recorded in our dataset. (Simultaneously coded by the two initial coders). Median grooming event length = **5 s** – 95th percentile = **19.10** ; **95.27%** of grooming events last less than 20s.

Supplementary Analysis 2: “UPSET / LOOK AT” behaviors in relation to grooming occurrence.

A two-way ANOVA revealed significant effects that varied according to the type of observation, indicating that the factors influenced the outcomes differently across behavioral categories F=24.45, p=3.41e-06; LOOK AT [5.87% of observation session duration (standard deviation 4.79%)] > UPSET [1.74% of observation session duration (standard deviation 2.85%)]. No effect of the presence or absence of grooming (F=2.00 - p=0.16), nor interaction effect (F=0.61 - p=0.44). Post hoc comparisons revealed a small but significant effect of [UPSET] (p = 0.02, below the Bonferroni-corrected threshold of 0.05/2). Specifically, [UPSET]

behavior was observed slightly less frequently during observation sessions that included grooming compared to sessions without grooming. In conclusion, LOOK AT and UPSET are events with very different probabilities of occurrence. LOOK AT, the child's action of looking at his/her parent, is a very frequent event whereas UPSET, the child getting angry and showing discomfort explicitly, is an event that was very rarely observed in our sessions.

We note that the latter is also observed even less when Grooming behavior is observed. It is possible that grooming alleviated anxiety, as the child stopped complaining when he/she started grooming. However, further research is needed to reach this conclusion.

Supplementary Analysis 3:

Probabilities of observing behaviors other than grooming were assessed immediately before and after the onset of a grooming event, allowing comparison of behavioral patterns surrounding grooming episodes. Then differences in the past vs post [grooming event] probability are compared using Fisher tests.

This analysis allows us to determine whether a grooming event alters the probability of observing other behaviors. For example, we can assess whether a grooming event is more likely to be preceded by the child looking at their parent ([LOOK AT]) than followed by it, or whether a grooming event decreases the likelihood of subsequent [UPSET] behavior. The results indicated no significant differences in the likelihood of observing any particular behavior before versus after the occurrence of a grooming event.

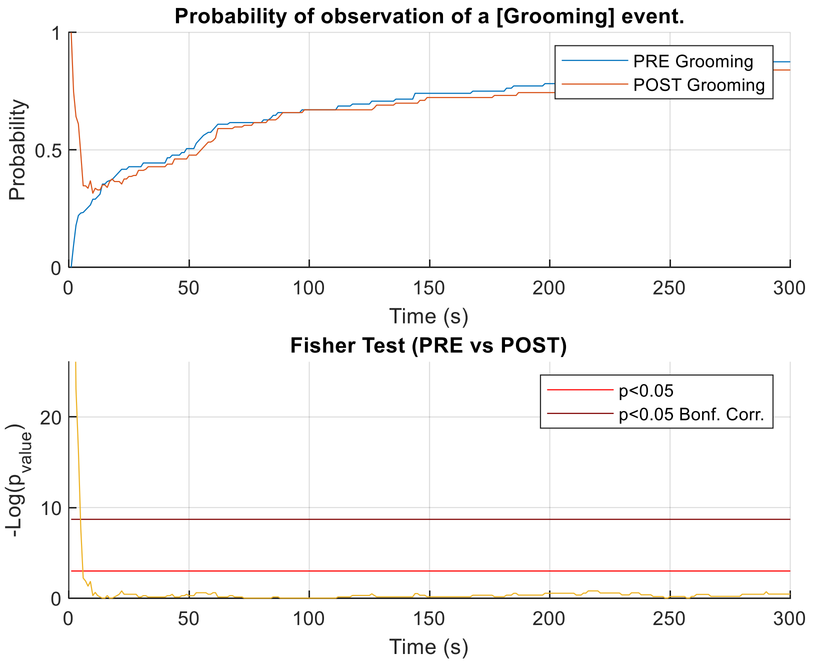

Supplementary Figure 5. The probability of observing a [GROOMING] event was assessed immediately before and after the occurrence of another [GROOMING] event to examine temporal patterns in grooming behavior.

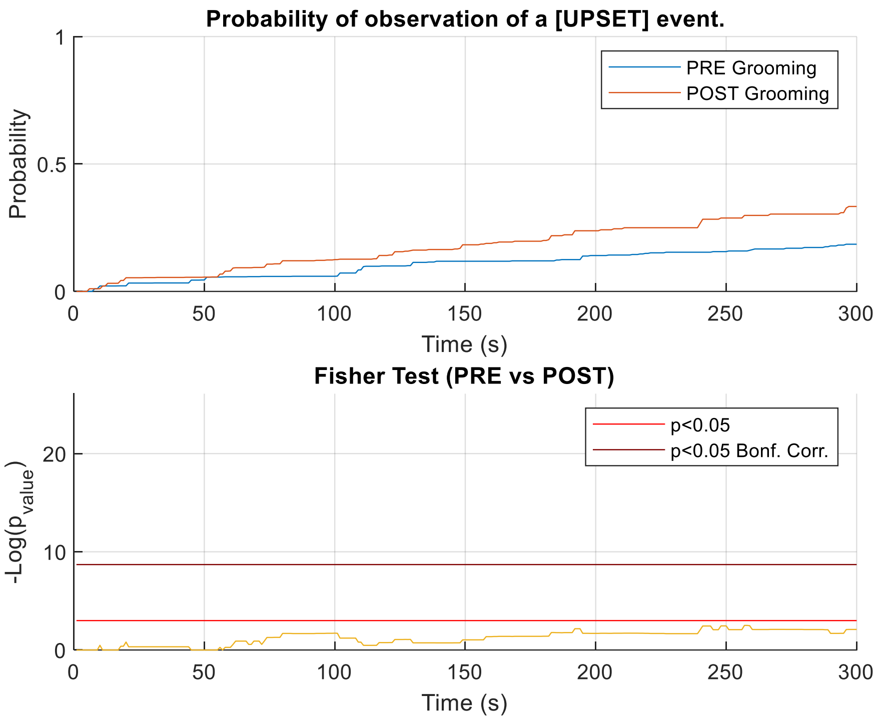

Supplementary Figure 6. Probability of observing an [UPSET] event immediately before and after a [GROOMING] event.

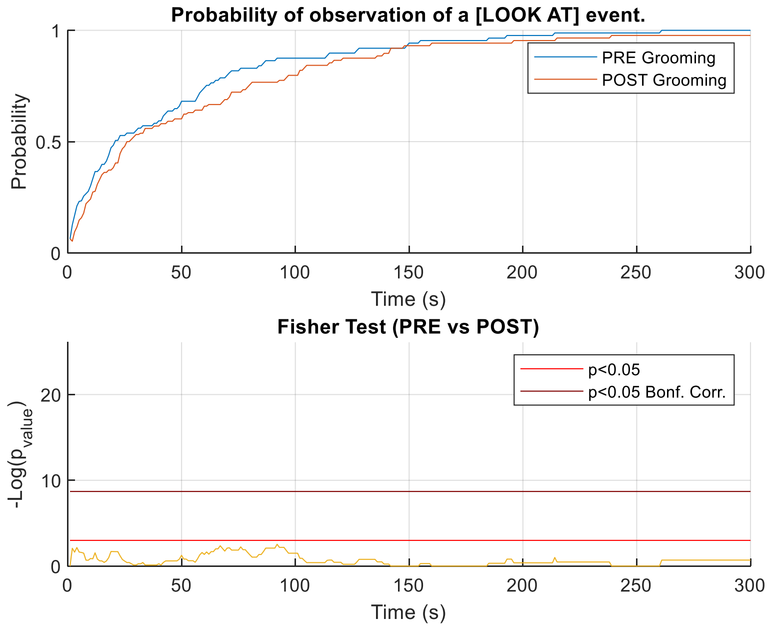

Supplementary Figure 7. Probability of observation of a [LOOK AT] event before and after a [GROOMING] event.

Supplementary Analysis 4: Reports from Primatologists

Objective

After evaluating video clips of infant behavior, 12 primatologists provided qualitative feedback on the key features they used to identify grooming gestures and distinguish them from other manual actions (e.g., grasping or holding). This analysis aimed to identify the critical behavioral features that experts rely on to evaluate the similarities and differences between infant and NHP grooming.

Methodology

Primatologists viewed video clips categorized as either "grooming present" or "grooming absent" following the evaluations by the two independent human coders. They were instructed to rate the similarity of the infants' behavior to NHP grooming on a 7-point Likert scale. After providing their scores, each expert was asked to complete an open-ended questionnaire designed to elicit the key behavioral features they used to assess similarity and to explain the differences they perceived between infant and NHP grooming.

The qualitative responses were analyzed using thematic analysis, focusing on the frequency and nature of the criteria reported by experts. Recurring concepts were grouped into key behavioral dimensions, and classified as the primary factors guiding the primatologists' evaluations of similarity.

Results

The analysis revealed that seven key behavioral dimensions underpinned the experts' assessments. These dimensions reflect the characteristics that define NHP grooming and were often cited as criteria for recognizing grooming and differentiating it from other manual behaviors.

1. Movement kinematics and precision: NHP grooming is characterized by precise, deliberate finger movements, often involving fine control of individual fingers. (Supplementary table 3)

2. Goal-Directedness: Grooming in NHPs is typically goal-directed, with the clear objective of removing debris, parasites, or dirt. Several primatologists noted that goal-directedness was a key factor in distinguishing grooming from other actions like grasping or playful hand movements. (Supplementary table 3)

3. Use of Gaze: In NHPs, the groomer typically directs their gaze toward the area being groomed. Several primatologists noted that gaze direction was an important cue for recognizing grooming behavior in infants. (Supplementary table 3)

4. Bilateral Hand Coordination: NHPs often engage in bilateral hand coordination during grooming, with both hands working in a coordinated fashion. This is especially true when the groomer uses one hand to stabilize the grooming surface while the other hand performs fine manipulations. (Supplementary table 4)

5. Oral Involvement: A hallmark of NHP grooming is the use of the mouth to extract and inspect debris removed from the fur. Primatologists frequently referenced this behavior as a distinguishing feature. (Supplementary table 4)

6. Repetition and Rhythmicity: Grooming in NHPs is often rhythmic, involving repetitive pinching, picking, and sweeping motions. Repetition provides clear evidence of sustained goal-directed action. (Supplementary table 4)

7. Attention seeking: Eight out of twelve primatologists report that, in this experimental context, seeking attention could be a trigger for the behavior. (Supplementary table 5)

Summary of Insights

The seven key features described above form the basis of the primatologists' evaluation framework for identifying grooming gestures. When infants exhibited behaviors that satisfied multiple criteria, for example, precise movements, bilateral hand coordination, clear goal-directed actions, and repetitive patterns, primatologists were more likely to rate the behavior as similar to NHP grooming. These criteria were not always met simultaneously. For instance, infants could exhibit rhythmic, repetitive actions, although their movements may have been exploratory or attention-seeking, rather than precise and goal-directed. Similarly, some infants may fail to maintain sustained gaze on the grooming target. These inconsistencies likely explain why primatologists assigned ratings reflecting partial but not complete similarity to NHP grooming.

Primatologists also recognized that grooming skills in NHPs are acquired through social observation and practice and depend on experience and motor development. While human infants may exhibit grooming-like behaviors, their level of proficiency is naturally limited by their stage of sensorimotor development. From this perspective, the observed differences between infant and NHP grooming may not be indicative of a lack of homology but rather a reflection of developmental immaturity.

Primatologists' Questionnaire:

1. **Main research on field study or laboratory study?**
2. **Which models of non-human primate you are mainly working with?**
3. **Please rate your familiarity with grooming behavior in Non-human primate (1 – 7).**
4. **During the evaluation, what aspect of the infants' behavior that make you consider the movement is similar to monkey grooming? [0=not at all to 3=mandatory]**

- *Precision of the motor pattern*
- *Goal-directedness*
- *Gaze and direction of attention*

5. **Do you take account others aspects of the infants' behavior to make you consider the movement is similar to monkey grooming?**

6. **During the evaluation, why do you think the infant starts grooming? or what do you think triggers the infant's grooming behavior?**

7. **In your observation, do you think the infants are using grooming to capture her/his mother's attention and communicate with her?**

8. **In your observation, do you think the emotional state (Positive/ Negative) of the infants related to their grooming behavior? If yes, what's the correlation?**

9. **Do you have some other opinions or suggestions about the task or your observation of the behaviors?**

Primatologists' evaluation: Results

Supplementary Table 2: Primatologists' evaluation. (evaluation 1,2&3 [field, NHP model, familiarity] and video-recorded sessions evaluations.).

ID	Research environment	Model	Grooming Familiarity [1 to 7]	Score (All)	Score (Grooming=1)	Score (Grooming=0)	Z-Score (Grooming=1)	Z-Score (Grooming=0)	AUC (%)
**1**	Lab.	Macaques	5	1.95 (1.30)	2.73 (1.43)	1.18 (0.44)	0.60 (1.09)	-0.59 (0.33)	86.27
**2**	Both	Macaques	6	4.80 (2.24)	6.26 (1.40)	3.37 (1.97)	0.65 (0.62)	-0.64 (0.88)	89.03
**3**	Both	Macaques	5	3.05 (2.34)	4.62 (2.22)	1.50 (1.09)	0.67 (0.95)	-0.66 (0.47))	87.51
**4**	Both	Macaques	7	3.56 (2.27)	5.26 (1.62)	1.89 (1.42)	0.75 (0.71)	-0.73 (0.62)	92.91
**5**	Both	Baboon	7	2.39 (1.61)	3.62 (1.43)	1.18 (0.44)	0.76 (0.78)	-0.75 (0.27)	95.47
**6**	Both	Macaques	7	2.97 (1.99)	4.55 (1.54)	1.42 (0.81)	0.79 (0.77)	-0.78 (0.41)	93.69
**7**	Field	Chimpanzee	7	2.04 (1.48)	2.83 (1.71)	1.26 (0.55)	0.53 (1.15)	-0.52 (0.37)	80.90
**8**	Both	Macaques/Chimpanzee	6	3.01 (1.71)	4.32 (1.34)	1.72 (0.83)	0.76 (0.78)	-0.75 (0.49)	93.71
**9**	Lab.	Macaques	4	2.79 (2.20)	4.36 (2.11)	1.26 (0.68)	0.71 (0.96)	-0.70 (0.31)	92.45
**10**	Lab.	Macaques	5	2.53 (1.92)	3.87 (1.84)	1.22 (0.72)	0.69 (0.96)	-0.68 (0.37)	90.16
**11**	Field	Baboon	7	3.11 (1.91)	4.47 (1.70)	1.78 (0.90)	0.71 (0.69)	-0.70 (0.47)	89.66
**12**	Field	Chimpanzee	6	3.21 (1.68)	4.36 (1.48)	2.09 (0.94)	0.68 (0.88)	-0.67 (0.56)	89.13

Mean Score and standard deviation of primatologists' evaluations for the 107 video-recorded sessions with hand movements, labeled as grooming or not. Primatologists' score was higher for session labeled as grooming [4.27 (0.96)] compared to those not grooming [1.66(0.62)] ; Difference [2.61(0.59)] ; Paired t-test $\mathbf{p_{value}=8.44x10^{-9}}$.

To reduce inter-individual variations on the 7point Lickert scale, scores were converted on Z-scores. Similar results were observed. Z-scores of primatologists' ratings on the 7-point Likert scale were higher for sessions labeled as containing grooming (0.69 ± 0.07) than for sessions without grooming (-0.68 ± 0.07), with a mean difference of 1.37 ± 0.15; Paired t-test $\mathbf{p_{value}=2.80x10^{-12}}$.

Supplementary Table 3: Primatologist's evaluations (evaluation 4 [movement precision, goal, gaze/attention])

ID	Research environment	Model	Precision of the motor movement	Goal-directedness	Gaze and direction of attention
**1**	Lab.	Macaques	1	0	0
**2**	Both	Macaques	1	0	0
**3**	Both	Macaques	1	0,5	0,5
**4**	Both	Macaques	3	1	2
**5**	Both	Baboon	1	3	2
**6**	Both	Macaques	1	3	2
**7**	Field	Chimpanzee	1	2	3
**8**	Both	Macaques/Chimpanzee	2	3	1
**9**	Lab.	Macaques	1	0	2
**10**	Lab.	Macaques	1	0	0
**11**	Field	Baboon	1	2	3
**12**	Field	Chimpanzee	1	2	2

Supplementary Table 4: Primatologist's evaluations (evaluation 5 [other aspects of similarity])

ID	Research environment	Model	Other aspects used to assess similarity with monkey grooming
**1**	Lab.	Macaques	With one hand or two hand movement - two is more grooming. Gesture and mouth movements.
**2**	Both	Macaques	No
**3**	Both	Macaques	Mouth movements
**4**	Both	Macaques	If tapping then not grooming
**5**	Both	Baboon	If have soft touch and if repeat the movement
**6**	Both	Macaques	if it end with mouth eating; If use the mouth to eat the skin; both hand and focus on the movement; if it's slapping then no.
**7**	Field	Chimpanzee	[4 to end] with picking and eating behavior
**8**	Both	Macaques/Chimpanzee	4- both hand and coordinate with eyes; 5-end with mouth eating; 6 -repeat the behavior
**9**	Lab.	Macaques	No
**10**	Lab.	Macaques	Sloping, biting
**11**	Field	Baboon	Social context, mouth movement
**12**	Field	Chimpanzee	If mouth and picking then give score 7

Supplementary Table 5: Primatologists' evaluations (evaluation 6 [events triggers of grooming])

ID	Research environment	Model	What triggered
**1**	Lab.	Macaques	When the baby feels frustrated, not careful clumsy, and needs attention.
**2**	Both	Macaques	Body part is close to the baby and the person by their side is familiar.
**3**	Both	Macaques	Ask for attention.
**4**	Both	Macaques	Ask for attention and reassure.
**5**	Both	Baboon	/
**6**	Both	Macaques	Wants to explore and curious; want to attract the attention.
**7**	Field	Chimpanzee	Attract attention/ Want to play with mother.
**8**	Both	Macaques/Chimpanzee	Capture the attention or explore the environment.
**9**	Lab.	Macaques	/
**10**	Lab.	Macaques	Attention seeking.
**11**	Field	Baboon	Physical contact bound; explore the skin and also when they are frustrated they might start to groom.
**12**	Field	Chimpanzee	Attract attention, explore skin.

Supplementary Table 6: Primatologists' evaluations judgement 7; (grooming for attention from caregiver).

ID	Research environment	Model	Capture attention / communication
**1**	Lab.	Macaques	In some cases, but less than half.
**2**	Both	Macaques	Most of the time it's exploring.
**3**	Both	Macaques	Yes
**4**	Both	Macaques	Some cases yes, but most of the time is because uncomfortable.
**5**	Both	Baboon	No opinion
**6**	Both	Macaques	Yes
**7**	Field	Chimpanzee	More attention, hard to say communication.
**8**	Both	Macaques/Chimpanzee	Yes, try to open the communication.
**9**	Lab.	Macaques	No opinion
**10**	Lab.	Macaques	Yes
**11**	Field	Baboon	Not really for communication.
**12**	Field	Chimpanzee	Yes

Supplementary Table 7: Primatologist’s evaluation (evaluation 8 [grooming and emotional states]).

ID	Research environment	Model	Emotion State
**1**	Lab.	Macaques	No
**2**	Both	Macaques	Depends, most of the time bored then grooming.
**3**	Both	Macaques	More chill more groom.
**4**	Both	Macaques	Could be, more uncomfortable/ negative emotion, more grooming.
**5**	Both	Baboon	No opinion
**6**	Both	Macaques	When they are happy they don't groom.
**7**	Field	Chimpanzee	Hard to say.
**8**	Both	Macaques/Chimpanzee	Before the grooming more negative less groom; after the grooming looks more positive and calm because focus.
**9**	Lab.	Macaques	No opinion
**10**	Lab.	Macaques	Depends, some seem to enjoy grooming, others seem to be anxious and attention-seeking.
**11**	Field	Baboon	Yes both might related to more grooming.
**12**	Field	Chimpanzee	Yes, more positive more groom.

Supplementary Table 8: Primatologists’ evaluations (evaluation 9 [general observations]

ID	Research environment	Model	Suggestions ?
**1**	Lab.	Macaques	Direction of camera. If it's skin grasping I don't consider it’s grooming, but hair touch more grooming.
**2**	Both	Macaques	No
**3**	Both	Macaques	No
**4**	Both	Macaques	Context of lab and home is different.
**5**	Both	Baboon	No
**6**	Both	Macaques	No
**7**	Field	Chimpanzee	Gender, Chimpanzee male need more grooming than female; hard to say language development, more focus on the point what they groom for, in a communication needs view.
**8**	Both	Macaques/Chimpanzee	Consider the grooming not only for the motor but also for the social need.
**9**	Lab.	Macaques	No
**10**	Lab.	Macaques	No
**11**	Field	Baboon	No
**12**	Field	Chimpanzee	No

Supplementary Analysis 5A : Longitudinal analysis of grooming observations compared to grasping observations.

Hypothesis :

The acquisition of fine motor skills in child development is typically assessed using a series of observational milestones often via object-reaching tasks. The palmar grasp emerges around 5 months, followed by the radial palmar grasp at approximately 7 months. The inferior pincer grasp generally appears around 9 months with the thumb-index pincer grasp developing between 10-12 months[1] . Interestingly, spontaneous thumb-index pincer grasp patterns can also be observed in children as early as 1 to 5 months, expressed without any goal-directed reaching movement[2].

Within the framework of our hypothesis on grooming suppression, we predicted the onset of pinch grip use for grooming at approximately 6–7 months, with its expression declining and ceasing by around 18 months of age. To examine this, we conducted a longitudinal analysis of the probability of observing grooming and grasping behaviors. No suppression was hypothesized for grasping; instead, we expected the probability of observation to remain stable or to increase as children engaged in the acquisition and refinement of fine motor skills

Analysis:

The relationship between the probability of observing grooming behavior and age was tested across subject longitudinally (N=17) using repeated measure correlation (rmcorr [39]) with the rmcorrShiny application[40] . Grasping and Grooming probabilities were compared with the actual age at the moment of observation, or with the ordering of within-subject observations (first, second, etc..). A complete report of the data and results of the analysis using the rmcorrShiny application can be found in the ReportResultsLongitudinal_rmcorrShiny.pdf document (https://osf.io/es3fn).

Results :

Longitudinal analysis: Grooming vs Age

Output from rmcorr
Repeated measures correlation: -0.441, Degrees of freedom: 34, p-value: 0.007
95% Confidence Interval: -0.672, -0.132; **r_{rm}(34) = -0.44**, 95% CI [-0.672, -0.132], **p = 0.007**

Longitudinal analysis : Grooming vs Order of observations.

Output from rmcorr

Repeated measures correlation: -0.453
Degrees of freedom: 34
p-value: 0.006
95% Confidence Interval: -0.68, -0.146

Reportable results
r_{rm}(34) = -0.45, 95% CI [-0.68, -0.146], **p = 0.006**

Longitudinal analysis : Grasping vs Age

Output from rmcorr

Repeated measures correlation: 0.308
Degrees of freedom: 34
p-value: 0.067
95% Confidence Interval: -0.023, 0.578

Reportable results

$r_{rm}(34) = 0.31$, 95% CI [-0.023, 0.578], $p = 0.067$

Longitudinal analysis : Grasping vs Order of observations.

Output from rmcorr

Repeated measures correlation: 0.318
Degrees of freedom: 34
p-value: 0.058
95% Confidence Interval: -0.011, 0.586

Reportable results

$r_{rm}(34) = 0.32$, 95% CI [-0.011, 0.586], $p = 0.058$

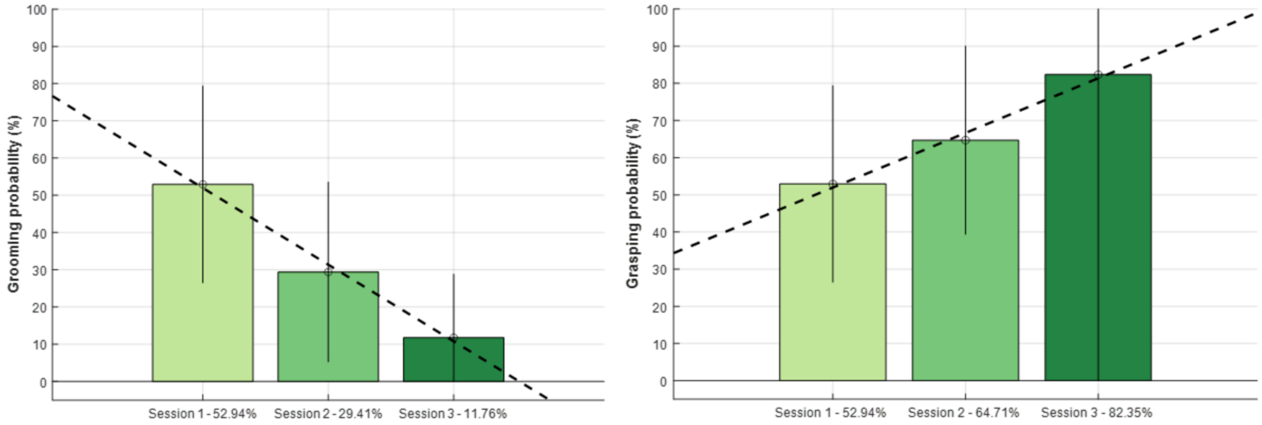

Supplementary Figure 8 : Results from the longitudinal analysis of Grooming (Left) and Grasping (Right). Longitudinal data for grooming (left) and grasping (right) indicate that only the negative correlation between grooming and age (Order of observations) reached significance, rrm(34) = –0.45, 95% CI [–0.68, –0.15], p = 0.006. By contrast, the correlation between grasping and age (Order of observations) did not reach significance, rrm(34) = 0.32, 95% CI [–0.01, 0.59], p = 0.058.

Conclusion :

Our results indicate that, within the observed time frame, the probability of observing grooming decreased significantly with age. In contrast, no comparable trend, nor a significant inverse association, was observed for grasping.

References :

1. Gerber, R. J., Wilks, T. & Erdie-Lalena, C. Developmental Milestones: Motor Development. *Pediatrics in Review* **31**, 267–277 (2010).

2. Wallace, P. S. & Whishaw, I. Q. Independent digit movements and precision grip patterns in 1–5-month-old human infants: hand-babbling, including vacuous then self-directed hand and digit movements, precedes targeted reaching. *Neuropsychologia* **41**, 1912–1918 (2003).

Supplementary Analysis 5b : GLMM analysis of grooming observations compared to grasping observations.

A fast GLMM analysis was conducted using all observations to examine the effect of age (~7–18 months) on the probability of observing grooming or grasping. Models were implemented in MATLAB (R2023b) using the fitglme function.

In the first model, which included critical period, gender, and environment as random effects, age was significantly negatively associated with grooming (coefficient = –0.04, $p = 0.0178$), while no significant association was observed for grasping (coefficient = 0.01, $p = 0.2428$).

In the second model, which additionally accounted for repeated measurements by including participant ID as a categorical random effect, the negative association between age and grooming remained significant (coefficient = –0.04, $p = 0.0060$), and again no significant effect was found for grasping (coefficient = 0.02, $p = 0.1820$). These results indicate that grooming behavior decreases with age, whereas grasping probability does not show a significant age-related change.

Conclusion :

Consistent with Supplementary Analysis 5A, which included only subjects observed longitudinally, the probability of observing grooming decreased over the observation period. In contrast, no comparable trend, nor a significant inverse association, was observed for grasping.

Supplementary Analysis 6 : Alternative analysis of the Mac-Arthur Bates CDI by contrasting Observations with and without grooming.

In the initial analysis, children observed longitudinally contributed multiple observations. Given that assessments were conducted at several-month intervals, we expected their CDI scores to increase with each subsequent evaluation. To address this, the distributed code includes an option to either (1) exclude all children with multiple observations or (2) consider only the first observation for each child.

Results **:**

A *Original results :*

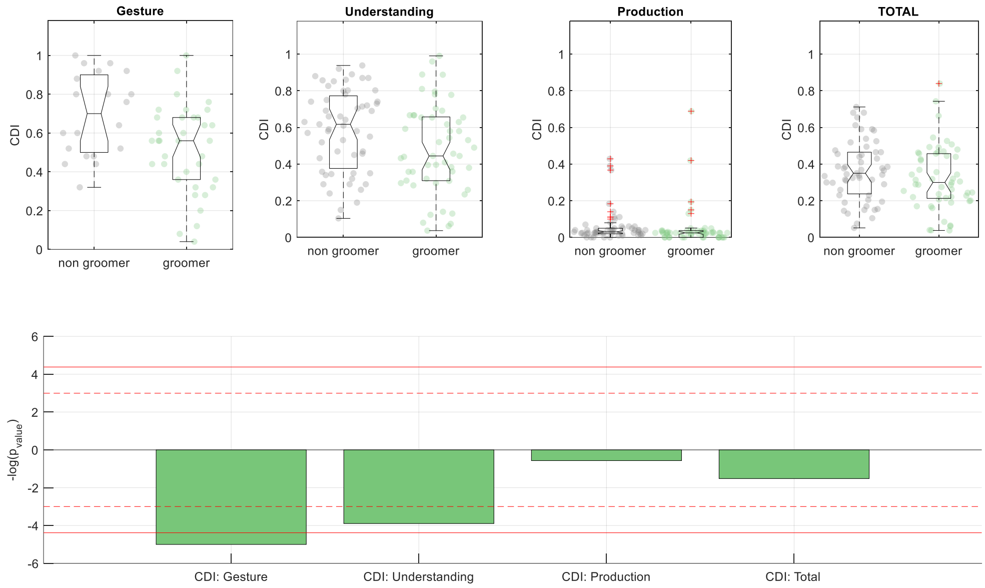

B *Results with exclusion of all children observed multiple times:*

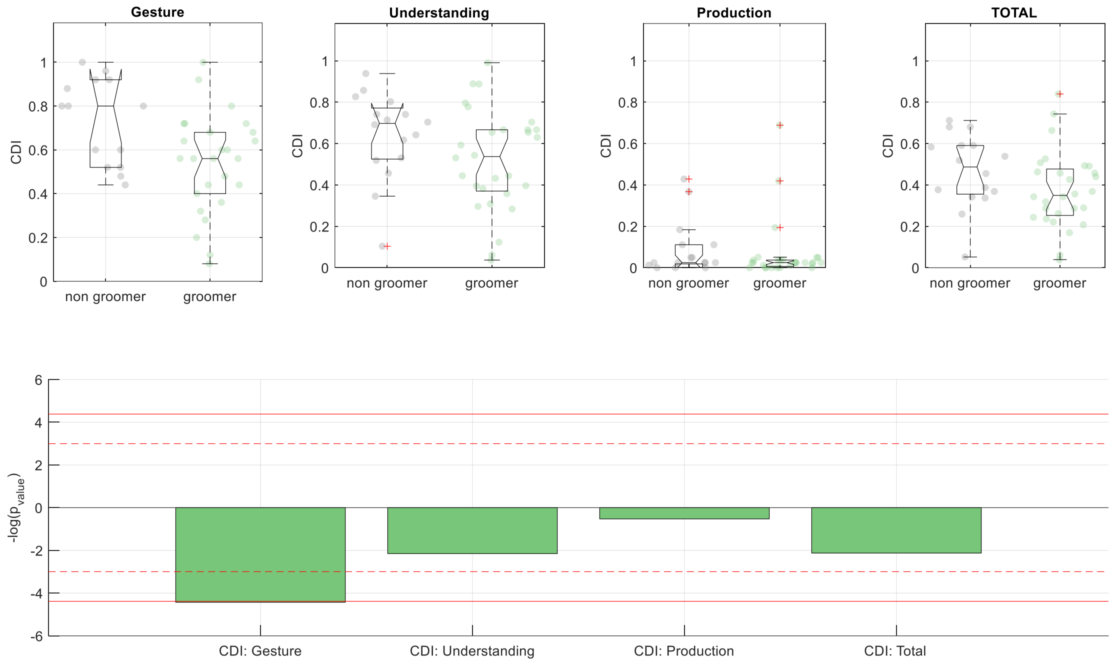

C *Results with only the first observation:*

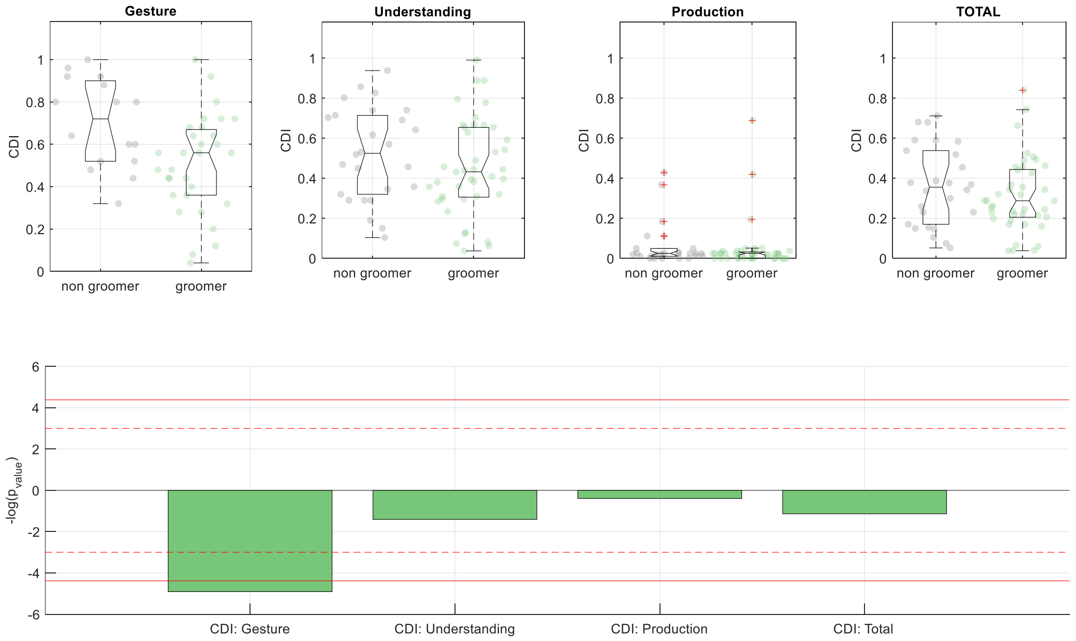

Supplementary Figure 9. Analyses of MacArthur–Bates CDI scores in relation to Grooming versus Non-Grooming observations. (**A**) Analysis including the full dataset; (**B)** Analysis excluding participants with longitudinal observations. (**C)** Analysis restricted to each participant's first observation.

The results are consistent with those reported in the original submission. In this population, primarily recruited between 7 and 18 months of age, only the CDI Gestures and CDI Understanding items captured meaningful neurodevelopmental variance. Notably, only the CDI Gestures measure demonstrated a robust difference between groomers and non-groomers.

Supplementary Analysis 7: General Linear Mixed Modeling of Grooming Probability

This analysis models the probability of observing grooming as a function of age, sex, experimental environment, and critical period of the day using a general linear mixed-effects model.

Hypothesis

The initial study identified several variables potentially influencing the probability of observing grooming behavior:

Age at observation: It was predicted that the probability of observing grooming would decrease with age, as this behavior tends to diminish in older children.

Time of day: The period of observation was considered potentially significant. The beta-endorphin hypothesis suggested an optimal observation window in the early afternoon, whereas the child's nap and sleep schedule predicted suboptimal periods at various times, including the evening. These effects could also interact with age.

Environment: The experimental context (laboratory vs. home) was expected to potentially influence behavior.

Sex/Gender: Included as a standard control variable in developmental studies.

As the existence and magnitude of these effects were not known a priori, we adopted a parsimonious approach, testing each variable independently and subsequently including only those with significant effects in multidimensional modeling. This strategy limits the number of tests by focusing on interaction effects likely to meaningfully influence the behavior of interest. Consequently, only age group and time of day were included in an ANOVA model, which revealed a decrease in grooming probability with age and an optimal observation period between 2:00 and 4:00 PM, with no significant interaction with age. However, this approach has the limitation of potentially overlooking minor effects that could be masked by stronger main effects.

Additionally, it has been suggested that a regression-based analysis would allow the use of actual age at observation rather than a categorical age grouping. Here, we test four alternative model specifications using the full set of variables, treating age as a continuous variable within Generalized Linear Mixed Models (GLMMs) and standard linear models, in order to evaluate all main and interaction effects while accounting for the critical observation period identified previously.

Software

GLMM modeling were implemented using the Matlab (R2023b) Generalized linear mixed-effects model class with the fitglme function. Linear modeling with interactions were implemented using the Matlab (R2023b) LinearModel class with the fitlm function.

Results

Alternative Modeling 1 :

Searching an optimal observation period during the day (critical period) using GLMM, [Age] and [Critical Period] as fixed-effects and [Environment] and [Sex/Gender] as random-effects. The best model is selected by minimizing the AIC (Aikake Information Criterion).

Equation : GroomingPresence ~ 1 + Age + CritPeriod + (1 | Gender) + (1 | Environment)

The best-fitting model identified an optimal observation period of one hour, between 3:00 and 4:00 PM, and revealed a negative relationship between age and the probability of observing grooming.

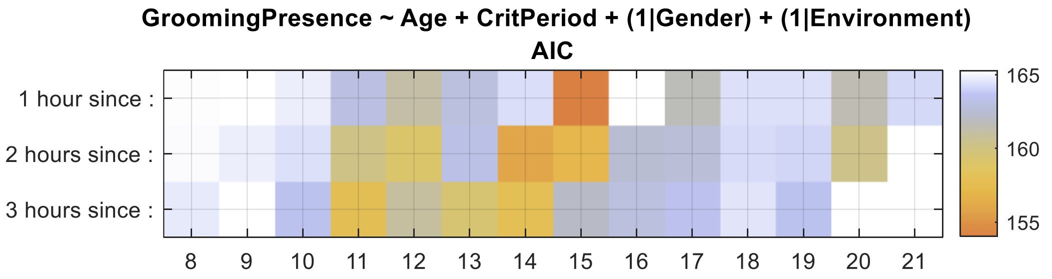

Supplementary Figure 10: AIC for all considered models/Gender and Environment as random effects.

Supplementary Table 9: Alternative Model 1 - fit statistics:

AIC	*BIC*	*LogLikelihood*	*Deviance*
154.03	170.12	-71.014	142.03

Supplementary Table 10: Alternative Model 1 - Fixed effects coefficients (95% CIs):

Name	*Estimate*	*SE*	*tStat*	*DF*	*pValue*	*Lower*	*Upper*
(Intercept)	0.80463	0.18495	4.3505	105	3.1578e-05	0.43791	1.1713
'Age'	**-0.03324**	0.014958	**-2.2227**	105	**0.028378**	-0.06291	-0.00359
'CritPeriod_true'	**0.36847**	0.10686	**3.4483**	105	**0.00081305**	0.15659	0.58034

Alternative Modeling 2 :

This analysis aimed to identify the optimal observation period during the day (critical period) using a GLMM, with **age**, **critical period**, **environment**, and **sex/gender** included as fixed effects. The best-fitting model was selected by minimizing the Akaike Information Criterion (AIC).

Equation : GroomingPresence ~ 1 + Age + CritPeriod + Gender + Environment

The best-fitting model identified an optimal observation period of one hour, between 3:00 and 4:00 PM, and revealed a negative relationship between age and the probability of observing grooming. No significant effects of environment or sex/gender were detected. The model with all variables included as fixed effects yielded the lowest AIC.

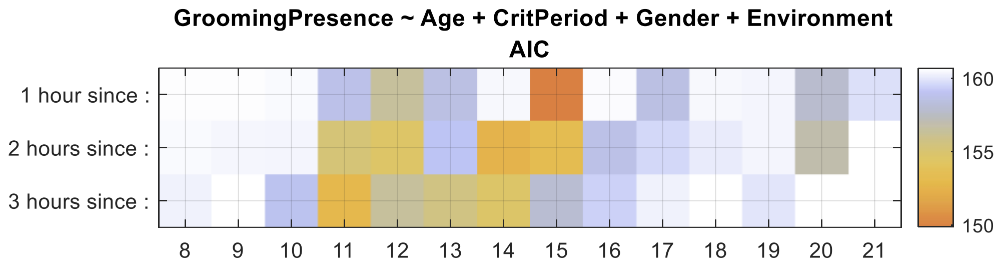

Supplementary Figure 11: AIC for all considered models /All variables as fixed effects.

Supplementary Table 11: Alternative Model 2 - fit statistics:

AIC	*BIC*	*LogLikelihood*	*Deviance*
149.89	165.98	-68.944	137.89

Supplementary Table 12: Alternative Model 2 - Fixed effects coefficients (95% CIs):

Name	*Estimate*	*SE*	*tStat*	*DF*	*pValue*	*Lower*	*Upper*
(Intercept)	0.76151	0.18897	4.0298	103	0.00010708	0.38673	1.1363
'Environment_Lab'	0.17656	0.090603	1.9487	103	0.054052	-0.00313	0.35625
'Gender_M'	0.041855	0.088825	0.47122	103	0.63848	-0.13431	0.21802
'Age'	**-0.037795**	0.014885	**-2.5392**	103	**0.012606**	-0.06731	-0.00827
'CritPeriod_true'	**0.35451**	0.10513	**3.3722**	103	**0.0010512**	0.14602	0.56301

Alternative Modeling 3 :

Linear regression model using the previously determined critical period: [Critical Period]=2pm to 4pl, [Age] as a groups (1,2 and 3), [Environment], [Sex/Gender] with interactions.

Supplementary Table 13: Alternative model 3 – Linear regression model coefficients with interactions.

	Estimate	*SE*	*tStat*	*pValue*
(Intercept)	0.51178	0.048609	10.529	9.7205e-18
Age group	**-0.11317**	**0.048418**	**-2.3374**	**0.021475**
Sex	0.036146	0.047781	0.7565	0.45118
Environment	0.076578	0.048726	1.5716	0.1193
2pm-4pm Crit. Period.	**0.15447**	**0.049395**	**3.1273**	**0.0023291**
Age group:Sex	0.014407	0.04902	0.2939	0.76946
Age group:Environment	-0.0035266	0.051672	-0.06825	0.94573
Age group:2pm-4pm Crit. Period.	-0.045501	0.051672	-0.89388	0.3736
Sex:Environment	0.0096778	0.049847	0.19415	0.84646
Sex:2pm-4pm Crit. Period.	0.067016	0.04887	1.3713	0.17344
Environment:2pm-4pm Crit. Period.	-0.035566	0.04886	-0.72791	0.46842

<u>Alternative Modeling 4 :</u>

Linear regression model using the previously determined critical period : [Critical Period]=2pm to 4pl, [Age] as a continuous variable, [Environment], [Sex/Gender] with interactions.

Supplementary Table 14: Alternative model 4 – Linear regression model coefficients with interactions.

	Estimate	***SE***	***tStat***	***pValue***
(Intercept)	0.50724	0.048647	10.427	1.6084e-17
Age	**-0.11211**	**0.050554**	**-2.2177**	**0.028908**
Sex	0.029758	0.047616	0.62496	0.53347
Environment	0.079529	0.048532	1.6387	0.10451
2pm-4pm Crit. Period.	**0.15125**	**0.049218**	**3.0731**	**0.0027504**
Age:Sex	0.028499	0.049962	0.5704	0.56972
Age:Environment	0.024708	0.05045	0.48976	0.62541
Age:2pm-4pm Crit. Period.	-0.032112	0.049775	-0.64513	0.52037
Sex:Environment	-0.0036676	0.049627	-0.073905	0.94124
Sex:2pm-4pm Crit. Period.	0.062527	0.048888	1.279	0.20396
Environment:2pm-4pm Crit. Period.	-0.036679	0.048866	-0.7506	0.45471

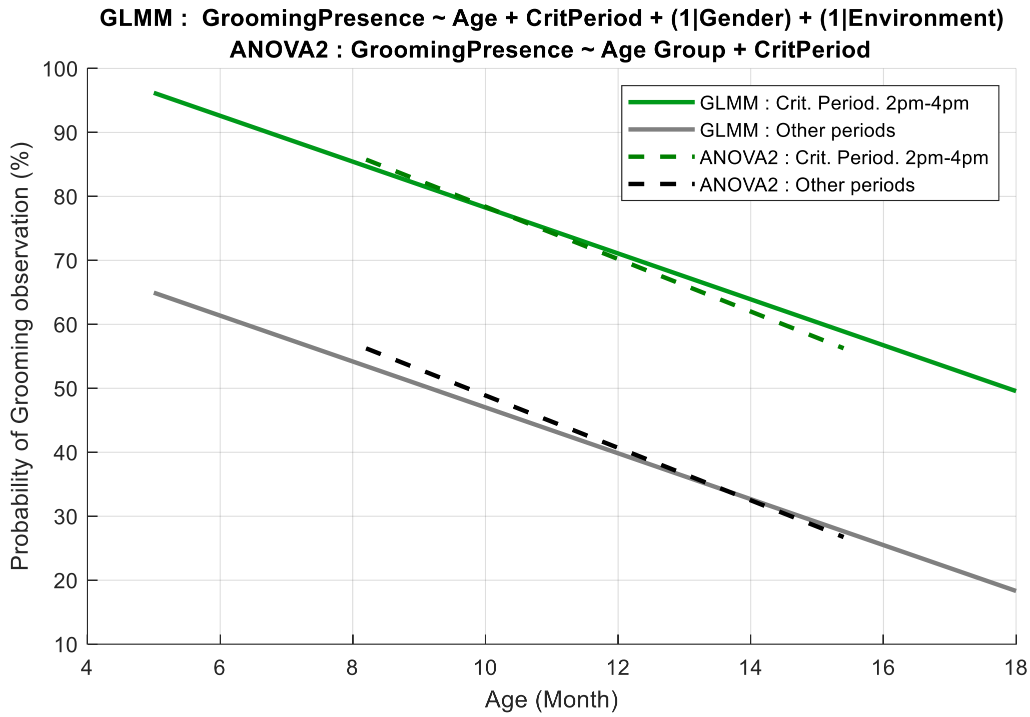

Supplementary Figure 12 : Prediction of grooming occurrence was performed using both the initial ANOVA2 model and a GLMM including age and the 2:00–4:00 PM critical period as fixed effects.

Conclusion:

All models consistently indicate two primary, independent effects: an optimal observation period in the early afternoon and a decreasing probability of observing grooming with age, regardless of whether age is modeled as a continuous variable or categorized into three successive age groups.

Supplementary Analysis 8: control conditions

We conducted two control conditions that were not included in the original manuscript, as they involved a limited number of infants. These control tasks were designed to assess whether grooming behavior depends on the identity of the grooming target. In the first condition, the ***stranger condition***, a stranger sat next to the infant while the parent stood in the room but remained out of the infant's view. Six infants were tested. None of them displayed grooming behavior toward the stranger's arm. Their responses varied: some stared at the stranger with apparent puzzlement and looked around, possibly searching for the parent; others became distressed and began crying, prompting early termination of the session. Due to these reactions, we decided not to continue this control condition.

In the second control condition, the ***fur condition***, we tested whether grooming could be elicited by any hairy stimulus. A faux-fur collar was placed in front of the infant in the same position typically occupied by the parent's arm. None of the infants tested (N=10) showed grooming behavior. Instead, the children stripped the faux fur from the tray of the chair and either handed it to the parent or threw it on the floor. These results are now reported in the Supplementary Information (see Supplementary analysis 8, table 1), although we emphasize that this point requires further investigation and should be interpreted with caution. Despite the limited sample size, the outcomes of these control tasks suggest that grooming behavior was specifically triggered by the presence of the parent, consistent with its proposed role in social bonding.

Supplementary Table 15: Control experiment: Fur condition.

ID	*Recording Place*	*Age (months)*	*Gender*	*Country*	*Grooming the parent*	*Grooming the fur*	*Throwing the fur*	*Grasping the fur*	*Passing to the parent*
1	Home	10,63	M	French	Yes	No	Yes	No	No
2	Home	12,00	M	French	Yes	No	Yes	Yes	Yes
3	Lab.	11,40	F	French	Yes	No	Yes	Yes	Yes
4	Lab.	15,70	M	French	Yes	No	Yes	Yes	Yes
5	Home	15,03	F	Chinese / French	Yes	No	Yes	Yes	No
6	Lab.	15,80	F	French	Yes	No	Yes	Yes	Yes
7	Home	14,77	M	Chinese	Yes	No	Yes	No	No
8	Home	11,50	F	Chinese	Yes	No	Yes	No	No
9	Lab.	8,13	F	French	Yes	No	Yes	Yes	Yes
10	Lab.	18,60	M	French	Yes	No	Yes	Yes	Yes
